# The SIRT5-JIP4 interaction promotes osteoclastogenesis by modulating RANKL-induced signaling transduction

**DOI:** 10.1186/s12964-024-02021-x

**Published:** 2025-01-14

**Authors:** Kecheng Zhu, Chunxiang Sheng, Linlin Zhang, Yuying Yang, Xiaojing Chen, Tao Jiang, Jiaxi Song, Deng Zhang, Xiao Wang, Hongyan Zhao, Lihao Sun, Libin Zhou, Bei Tao, Jianmin Liu

**Affiliations:** 1 Department of Endocrine and Metabolic Diseases, Shanghai Institute of Endocrine and Metabolic Diseases, Ruijin Hospital, Shanghai Jiao Tong University School of Medicine, 197 Ruijin Road II, Shanghai, 200025 China; 2 Shanghai National Clinical Research Center for Metabolic Diseases, Key Laboratory for Endocrine and Metabolic Diseases of the National Health Commission of the PR China, Shanghai Key Laboratory for Endocrine Tumor, State Key Laboratory of Medical Genomics, Ruijin Hospital, Shanghai Jiao Tong University School of Medicine, Shanghai, China

**Keywords:** SIRT5, JIP4, Osteoclast differentiation, Osteoporosis, RANKL, MAPK

## Abstract

Receptor activator of nuclear factor kappa-B ligand (RANKL) initiates a complex signaling cascade that is crucial for inducing osteoclast differentiation and activation. RANKL-induced signaling has been analyzed in detail, and the involvement of TNF receptor-associated factor 6 (TRAF6), calmodulin-dependent protein kinase (CaMK), NF-κB, mitogen-activated protein kinase (MAPK), activator protein-1 (AP-1), and molecules that contain an immunoreceptor tyrosine-based activation motif (ITAM) has been reported. However, the precise molecular steps that regulate RANKL signaling remain largely unknown. Here, we revealed the indispensable role of a class III histone deacetylase (SIRT5) in the processes of RANKL-induced osteoclast differentiation and activation. SIRT5 expression in osteoclasts was increased during osteoclastogenesis upon stimulation with RANKL. The RANKL-induced signaling activation was suppressed in SIRT5-deficient osteoclasts but enhanced by SIRT5 overexpression. Mice with global or conditional monocytic lineage knockout of SIRT5 had increased bone mass and reduced osteoclast numbers. In the cytoplasm, SIRT5 interacted with the scaffold protein JNK-interacting protein 4 (JIP4) to finely regulate MAPK signaling, which was critical for osteoclast differentiation and activation. Pharmacological inhibition of the catalytic activity of SIRT5 effectively reversed bone loss in ovariectomized mice. Taken together, the results of this study reveal that the SIRT5-JIP4 axis is a novel positive regulator that finely regulates RANKL-induced osteoclast differentiation and suggest that targeting this axis is a therapeutic strategy for preventing osteoporotic bone loss.

**Graphical Abstract:**

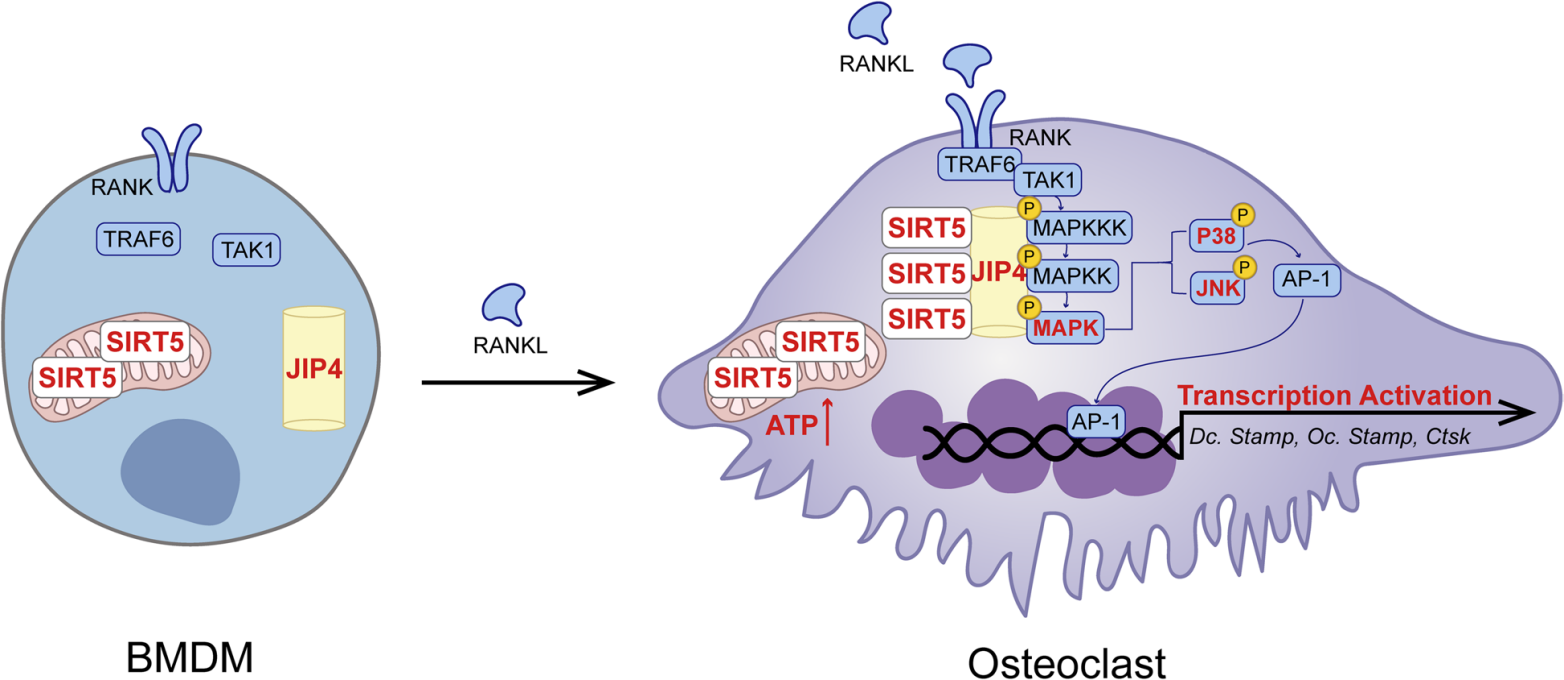

**Supplementary Information:**

The online version contains supplementary material available at 10.1186/s12964-024-02021-x.

## Introduction

The maintenance of bone homeostasis relies on the delicate balance of the activities of bone-resorbing osteoclasts and bone-forming osteoblasts [1]. Abnormal bone resorption by osteoclasts leads to bone destruction and is a hallmark of bone-related diseases such as osteoporosis [2]. Therefore, in-depth investigations of the molecular signaling and genetic programs underlying osteoclast differentiation and function will improve our understanding of osteoclast biology and provide a molecular basis for designing therapeutic strategies for bone remodeling diseases.

Osteoclasts are tissue-specific macrophage polykaryons that are generated by the differentiation of monocyte/macrophage precursor cells at or near the bone surface [3]. Osteoclastogenesis is coordinated by the binding of macrophage colony-stimulating factor (M-CSF) and receptor activator of nuclear factor kappa-B ligand (RANKL) to their receptors, namely, M-CSFR and RANK, respectively, on the surfaces of osteoclast precursors [4]. When RANKL binds to RANK, the cytoplasmic tail of RANK recruits the adaptor protein TNF receptor-associated factor 6 (TRAF6) to submembrane compartments, initiating a cascade of intracellular signaling events [5]. This binding triggers the activation of nuclear factor kappa-B (NF-κB) and calcium/calmodulin-dependent protein kinase (CaMK), both of which are essential for osteoclast function [6]. Additionally, immunoreceptor tyrosine-based activation motif (ITAM)-containing adaptors, such as Fc receptor common γ subunit (FcRγ) and DNAX-activating protein 12 (DAP12), provide costimulatory signals that synergize with RANKL-mediated pathways [7]. Among the pathways downstream of TRAF6, the mitogen-activated protein kinase (MAPK) pathway, which is initiated by the complex of TRAF6 and TGF-β-activated kinase (TAK1), plays a central role [8]. The pathway includes extracellular signaling-regulated kinases (ERKs), c-Jun N-terminal kinases (JNKs), and p38 MAPKs [9], which are crucial for osteoclast differentiation and activation. Specifically, the activation of JNKs and p38 MAPKs by RANKL induces the phosphorylation of c-Jun, an essential component of the activator protein-1 (AP-1) complex in osteoclasts, thereby increasing the expression of genes that are crucial for osteoclastogenesis [10].

Due to the thorough study and detailed mapping of the MAPK signaling pathway, it has become clear that a number of scaffold proteins play crucial roles in regulating this signaling network. Scaffold proteins are defined by the binding of at least two members of a signaling cascade, which bring together and organize components of the cascade to facilitate MAPK activation [11]. One study showed that the scaffold protein postsynaptic density protein 95 (PSD-95) is involved in osteoclast differentiation through association with immunoglobulin superfamily member 11 (IgSF11) [12]. Additionally, it has been demonstrated that receptor for activated C-kinase 1 (RACK1), another scaffolding protein, mediates the RANKL-dependent activation of p38 MAPK in osteoclast precursors and interacts with c-Src to regulate osteoclast function [13]. However, the precise roles of specific scaffold proteins in mediating MAPK signaling within the RANKL-RANK system in osteoclastogenesis have not yet been definitively elucidated.

SIRT5, an NAD^+^-dependent lysine deacetylase, is localized primarily to the mitochondrial matrix and most often modifies lysine succinylation, malonylation, and glutarylation to impact multiple enzymes that are involved in diverse mitochondrial metabolic pathways, thus regulating mitochondrial energy metabolism [14–16]. Osteoclastogenesis is an energy-consuming process supported by high metabolic activity. A substantial increase in the number of mitochondria during human osteoclast differentiation has been reported [17]. Compared with immature cells, fully differentiated osteoclasts have higher levels of enzymes in the electron transport chain and a higher mitochondrial oxygen consumption rate [17]. Given the pivotal role of energy metabolism in osteoclastogenesis, it is conceivable that SIRT5 may play an important regulatory role in this process. In addition, several recent studies have indicated that SIRT5 participates in various cellular differentiation processes [18, 19]. Considering the important role of SIRT5 in mitochondrial metabolism and cellular differentiation, further investigation into its role in osteoclast differentiation is warranted.

In the present study, we showed that SIRT5 positively regulates osteoclast differentiation by interacting with JNK-interacting protein 4 (JIP4), a scaffold protein with multiple protein interaction domains. We report here that RANKL signaling significantly increases SIRT5 levels in the cytoplasm during osteoclastogenesis. Deletion of the *Sirt5* gene results in increased bone mass and bone strength due to reduced osteoclast differentiation and activation. Through in vitro osteoclast culture systems, we demonstrated that SIRT5 forms a complex with JIP4 to finely modulate the RANKL-MAPK signaling cascade. Moreover, lentivirus-mediated knockdown of JIP4 inhibits osteoclast differentiation, suggesting a pivotal role of JIP4 in this process. Furthermore, pharmacological inhibition of the catalytic activity of SIRT5 effectively reversed bone loss in ovariectomized mice. Collectively, these results reveal the mechanism by which SIRT5 controls osteoclast differentiation and activation through its association with JIP4 to finely regulate the RANKL-RANK-MAPK system during osteoclast differentiation.

## Methods

### Mice

*Sirt5*-knockout (*Sirt5*^−/−^) mice were kindly provided by Professor Hongxiu Yu (Institutes of Biomedical Sciences, Fudan University, Shanghai, China). The *Sirt5*^floxed^ (Stock No. T013018) C57BL/6 breeding pairs were purchased from GemPharmatech CO, Ltd (Nanjing, China). *Lyz2*-cre C57BL/6 mice (Stock No.004781) were purchased from the Jackson Laboratory (Bar Harbor, USA). We generated a monocytic lineage-specific *Sirt5* knockout mouse model, *Sirt5*^Lyz2−/−^ mice, by crossing *Sirt5*^flox/flox^ mice with *Lyz2*-cre mice. *Sirt5*^flox/flox^ mice were used as the littermate controls. To study the effects of SIRT5 on ovariectomy (OVX)-induced bone loss in mice, female C57BL/6 mice were ovariectomized at 8 weeks of age. The control group underwent a sham operation. As shown in Fig. 5A, the female mice were randomly divided into 3 groups (*n* = 8 per group): the sham model (sham) group, the OVX model (OVX) group, the experimental OVX plus NRD167(Selleck Chemicals, Houston, USA) injection (OVX + NRD167) group. One weeks after surgery, the mice from the sham and the OVX groups were injected with vehicle, and the mice from the OVX + NRD167 groups were injected with NRD167 dissolved in vehicle. The injections were administered every other day. The vehicle is consisted of 10% DMSO, 40% PEG300, 5% Tween-80 and 5% saline. Six weeks after surgery, the mice were euthanized. All the mice were housed in a Specific-Pathogen-Free environment under 12 h–12 h light-dark cycles with *ad libitum* feeding. All experimental animal protocols were performed in accordance with the ARRIVE guidelines and Institutional Animal Care and Use Committee (IACUC) guidelines and approved by the IACUC of Shanghai Model Organisms Center (2019-0026).

### Micro-CT analysis

The right femur from each mouse was extracted and fixed in 4% paraformaldehyde for 48 h. Subsequently, the femurs were stored long-term in 70% ethanol. All femurs were scanned using SkyScan 1176 (Bruker, Kontich, Belgium) at an 8 μm resolution and quantitatively analyzed following the guidelines of the American Society for Bone and Mineral Research [20]. Trabecular bone analysis of the distal femur was performed in the region of 224 slices immediately adjacent to the growth plate. For cortical bone analysis, the region between 450 and 516 slices above the growth plate was examined. CTAn analysis software was used to determine all microstructural parameters. Additionally, three-dimensional (3D) images were reconstructed based on the two-dimensional (2D) scanned slices using CTvox software.

### Three-point bending test

For biomechanical testing, the left femurs were extracted and immediately subjected to a three-point bending test using the Instron 5569 mechanical-testing machine (Instron, Inc., Norwood, USA). The femurs were placed on two supports positioned 6 mm apart, and a load was applied at a rate of 1 mm/min until the bone fractured. Throughout the bending test, the computerized data acquisition system collected load-displacement data at a sampling rate of 100 Hz. Force-deflection curves were generated using a custom program (MATLAB; MathWorks Inc., Natick, MA, USA) to calculate biomechanical parameters such as maximum load, elastic modulus, energy to failure, and yield load.

### Bone histomorphometry

The femurs of the mice underwent preparation for bone histomorphometric analysis as follows: First, the femurs were cleared of soft tissues, then fixed in 4% paraformaldehyde for 24 h at 4℃, and subsequently subjected to decalcification in 23% ethylenediaminetetraacetic acid (EDTA) at 4℃ for a duration of 3 days. Afterward, the femurs were embedded in paraffin. Serial sections were stained using hematoxylin and eosin (HE) as well as tartrate-resistant acid phosphatase (TRAP) staining with a leukocyte acid phosphatase staining kit (Sigma-Aldrich). For histomorphometric analysis, a Leica DMI-3000B microscope equipped with a digital camera was employed. A specific region within the trabecular area of the femurs, located beneath the growth plate, was subjected to measurement. Osteoclasts were identified as TRAP-positive cells, and the osteoclast number per bone trabecular parameter (N.Oc/B.Pm) was calculated using IPP software on the TRAP-stained images. The osteoclast surface relative to the bone trabecular surface (Oc.S/BS) represented the ratio of the TRAP-stained area to the total bone area, and this was quantified utilizing IPP software.

For histomorphometry, 8-week-old male mice were injected intraperitoneally with 10 mg/kg calcein (1 mg/ml in 2% NaHCO3 solution, Sigma) and 40 mg/kg alizarin red S (2 mg/ml in H2O, Sigma) at day 9 and day 2 before euthanasia separately. The left femurs were fixed, dehydrated, and embedded in methyl methacrylate for hard tissue section at 4 μm thickness. The fluorescence-labeled images were taken by LSM 880 confocal microscope (Zeiss, Oberkochen, Germany).

### Measurement of serum biomarkers

Mice were fasted for at least 6 h. Following this, blood samples were collected, allowed to coagulate at room temperature for 1 h, and then centrifuged at 3000 rpm for 10 min. The resulting serum samples were divided into aliquots and stored at −80℃. ELISA assays were conducted to determine the levels of serum markers, including N-terminal propeptide of type I procollagen (PINP) (USCN Life Science, Wuhan, China), osteocalcin (OCN) (USCN Life Science, Wuhan, China), C-terminal telopeptide of type I collagen (CTX-I) (Immunodiagnostic Systems, Tyne & Wear, UK), RANKL (R&D Systems), and osteoprotegerin (OPG) (R&D Systems), following the manufacturer’s instructions.

### BMDM culture and lentivirus infection

Male C57BL/6 mice (4–8 weeks old; obtained from the Vital River Laboratory Animal Technology Co., Ltd., Shanghai, China) were euthanized to prepare bone marrow-derived macrophages (BMDMs). BMDMs were isolated as previously described [21]. Briefly, bone marrow cells extracted from the femurs and tibias were rinsed with α-MEM. Subsequently, they were placed in α-MEM supplemented with 10% FBS and 10 ng/mL macrophage colony-stimulating factor (MCSF; obtained from Sino Biological, Shanghai, China) for a 24-hour incubation period. Afterward, the nonadherent cells were collected and cultured in α-MEM containing 10% FBS, 1% antibiotic penicillin-streptomycin combination, and 20 ng/mL MCSF for 4 days to cultivate BMDMs. All cells were maintained in a humidified environment with 5% CO2 at 37℃.

Lentivirus-based SIRT5 or JIP4 short hairpin RNA (shRNA), lentivirus expressing mouse SIRT5 and relative controls were purchased from GeneChem (Shanghai, China). Lentivirus was transfected into BMDMs at a multiplicity of infection (MOI) of 40. After 48 h, 0.5 µg/ml puromycin was added into culture medium to sustain the knockdown of SIRT5 or JIP4 and the overexpression of SIRT5 during subsequent osteoclastic induction.

### Osteoclastogenic induction and TRAP staining

To cultivate fully mature osteoclasts, BMDMs were cultured in the presence of 50 ng/mL RANKL (R&D Systems) and 20 ng/mL MCSF (R&D Systems) for a duration spanning 3 to 5 days. Following differentiation, cells were subjected to TRAP staining using a leukocyte acid phosphatase kit from Sigma-Aldrich, following the manufacturer’s instructions. Osteoclasts were identified as TRAP-positive multinucleated cells containing three or more nuclei. These osteoclasts were subsequently visualized, captured in photographs, and quantified using an Olympus microscope.

### RNA isolation and qRT-PCR

Total RNA was extracted using Total RNA Extraction Reagent (Vazyme, Nanjing, China) and reverse transcribed into cDNA by PrimeScript™ RT Master Mix (Takara, Shiga, Japan). qRT-PCR was performed with QuantStudio™Dx Real-Time PCR Instrument (Applied Biosystems, Foster City, USA) using ChamQ Universal SYBR qPCR Master Mix (Vazyme) for quantifying the relative expression of genes. Primer sequences involved in qRT-PCR are described in Table S5. Gene expression was normalized to beta-actin in each sample.

### Western blot

Total protein was isolated using RIPA lysis buffer (Biocolors, Shanghai, China), and complemented with Protease Inhibitor Cocktail (APExBIO, Houston, USA). The denatured protein lysates were loaded onto SDS-PAGE gels for electrophoresis and subsequently transferred to PVDF membranes for primary antibody and horseradish peroxidase (HRP)-conjugated secondary antibody incubations. Immunoblotting signals were detected using ECL Luminescence Reagent (Meilunbio in Dalian, China), and visualized with the LAS-4000 Super CCD Remote Control Science Imaging System (Fujifilm, Tokyo, Japan). The specific antibodies used in this study are detailed in Table S6.

### Bone resorption assay

BMDMs were plated in 24-well dishes coated with synthetic carbonate apatite supplied by Cosmo BIO Co., Ltd. based in Tokyo, Japan. Osteoclastogenesis was initiated in *Sirt5* knockdown BMDMs with the presence of 50 ng/mL RANKL and 20 ng/mL MCSF. On the fifth day, the culture medium was aspirated from each well, and a 5% sodium hypochlorite treatment was applied for 5 min. After rinsing the plates with water and subsequent drying, designated regions within each well were captured using a microscope. The bone resorption rate was calculated as the proportion of bone resorption area relative to the overall carbonate apatite area, which was measured with Image-Pro Plus software (Media Cybernetics, Silver Spring, MD, USA).

### Glucose consumption assays and ATP measurements

For glucose consumption measurement, the media cultured BMDMs cells for 24 h were assayed with Glucose (HK) Assay Kit (Sigma, GAHK20) under the instructions and was corrected for total protein amount.

After 2 days of MCSF and RANKL treatment, BMDMs were plated into six-well plates at a density of 6 × 10^5^ cells per well. After 24 h, the ATP concentration in the cell lysate was measured with ATP Assay Kit (Beyotime, S0026) referred to the instructions attached to the kit.

### Oxygen consumption rate (OCR) and extracellular acidification rate (ECAR) measurements

For OCR measurement, BMDMs were treated with MCSF alone or with both MCSF and RANKL for 2 days, then seeded in an XF96 V3 microplate (Seahorse Bioscience) at a density of 3 × 10^4^ cells per well. OCR was measured using an XFe96 analyzer (Seahorse Bioscience) following the manufacturer’s instructions. Briefly, the cells’ culture medium was changed to Seahorse XF base medium containing 25 mM D-glucose, 2 mM sodium pyruvate, and 2 mM L-Glutamine and then incubated at 37 °C in a non-CO_2_ incubator (Seahorse Bioscience) for 1 h. Respiratory inhibitors (Seahorse Bioscience, 103015-100) were loaded into the injection port to reach the final concentrations of 1 µM oligomycin, 2 µM FCCP, 0.5 µM antimycin A, and 0.5 µM rotenone to detect uncoupled respiration, maximal respiration, and non-mitochondrial respiration, respectively. The final OCR results were normalized to total protein.

For ECAR measurement, cells were seeded in an XF96 V3 microplate (Seahorse Bioscience) coated with poly-L-lysine. ECAR was measured using an XFe96 analyzer (Seahorse Bioscience) following the manufacturer’s instructions. In brief, the culture medium of the cells was changed to Seahorse XF base medium with 2 mM L-Glutamine and incubated at 37 °C in a non-CO_2_ incubator (Seahorse Bioscience) for 1 h. Reagents were loaded into the injection port in sequence to reach the final concentration of 10mM glucose, 2 μm oligomycin and 0.1 M 2-Deoxy-D-glucose (2-DG). The final ECAR results were normalized to total protein.

### Immunoprecipitation (IP) and mass spectrum (MS)

BMDMs were transfected with lentivirus-based *Sirt5* short hairpin RNA (shRNA), lentivirus expressing mouse SIRT5 or relative controls for 24 h, and then were cultured in osteoclastogenic medium for 2 d. The cells were harvested and homogenized by sonication in IP lysis buffer (Beyotime Biotechnology). Lysates were incubated with either IgG, SIRT5, or JIP4 antibody for 2 h and then with protein A/G-agarose beads (Santa Cruz, Dallas, USA) overnight at 4 °C. The immunoprecipitated complexes were degenerated with SDS loading buffer for following standard western blot subsequent standard Western blot analysis. For IP-MS, the immunoprecipitated proteins underwent SDS-PAGE gel electrophoresis and were subsequently stained with brilliant Coomassie blue. The enriched proteins were then subjected to mass spectrometry analysis.

### Immunofluorescence (IF) staining

8 × 10^4^ BMDMs were plated in EZ Slides and incubated in α-MEM with or without RANKL for 24 h. The cells were immobilized by 10% formalin for 20 min, followed by permeabilization using 0.1% Triton X-100 for 5 min. Subsequent to a 1-hour blocking step in 5% BSA, the cells were subjected to an overnight incubation at 4°C with primary antibodies and detected using fluorescein-labeled secondary antibodies. Digital images were captured by LSM 880 confocal microscope (Zeiss, Oberkochen, Germany).

### Statistical analysis

The data is presented as mean ± standard deviation (SD). Statistical significance was assessed using either an unpaired Student’s t-test with a two-tailed distribution for two groups or ANOVA for multiple groups. Differences were considered to be statistically significant when *p* < 0.05.

## Results

### *Sirt5* knockout leads to increased bone mass and bone strength in mice

**Fig. 1:**
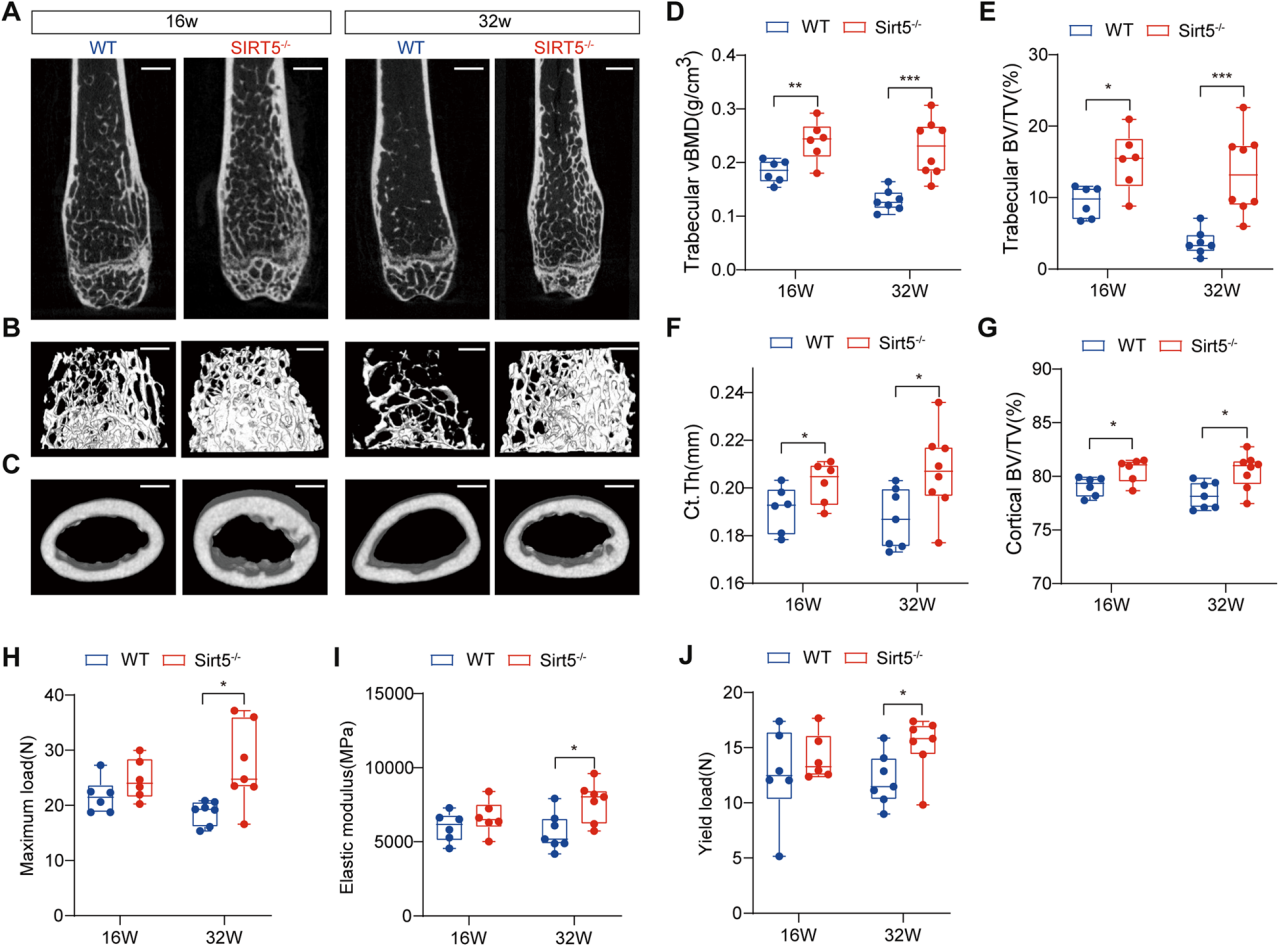
Knockout of *Sirt5 * leads to increased bone mass and bone strength in mice. **A**-**C** Representative 2D and 3D image reconstruction of coronal sections (**A**, scale bar, 800 μm), trabecular bone (**B**, scale bar, 500 μm) and cortical bone (**C**, scale bar, 600 μm) of distal femurs from male WT and *Sirt5*^−/−^ mice at 16 and 32 weeks of age. **D**-**G** Quantitative analysis of trabecular volumetric bone mineral density (vBMD) (**D**), trabecular bone volume per tissue volume (BV/TV) (**E**), cortical thickness (Ct.Th) (**F**) and cortical BV/TV (**G**) by microCT. **H**-**J** The maximum load (**H**), elastic modules (**I**) and yield load (**J**) of femurs from male WT and *Sirt5*^−/−^ mice at 16 and 32 weeks of age measured by three-point bending test. The data are presented as means ± SD (*n* = 7–8). ^*^p < 0.05, ^**^p < 0.01, ^***^p < 0.001 vs. WT

We first examined the bone characteristics of *Sirt5*-knockout (*Sirt5*^−/−^) mice. Micro-CT analysis was performed on femurs from 16-week-old and 32-week-old WT and *Sirt5*^−/−^ mice. As shown in Fig. 1A-C, *Sirt5* knockout led to increases in trabecular bone mass and cortical bone thickness. In addition, the microstructure of the distal femurs of *Sirt5*^−/−^ mice revealed elevated trabecular volume bone mineral density (vBMD), trabecular and cortical volume per tissue volume (BV/TV), and cortical thickness (Ct.Th) (Fig. 1D-G). Additionally, *Sirt5*^−/−^ mice presented increases in the trabecular surface area density (BS/TV), trabecular number (Tb.N), and cortical BV/TV (Tables S1 and S2). Moreover, *Sirt5*^−/−^ mice presented a reduction in trabecular separation (Tb.Sp), the trabecular pattern coefficient (Tb.Pf), and the structural model coefficient (SMI) of trabecular bone (Tables S1 and S2).

A three-point bending test was further performed to assess the mechanical properties of femurs from WT and *Sirt5*^−/−^ mice. No significant changes in the biomechanical parameters of the *Sirt5*^−/−^ mice were observed at 16 weeks of age (Fig. 1H-J). However, at 32 weeks of age, the maximum load (Fig. 1H), elastic modulus (Fig. 1I), and yield load (Fig. 1J) of the *Sirt5*^−/−^ mice were significantly greater than those of the WT mice. These findings suggest that ablation of *Sirt5* increases bone strength and resistance to fractures in mice.

### Osteoclast number and activity are reduced in *Sirt5*^−/−^ mice

**Fig. 2:**
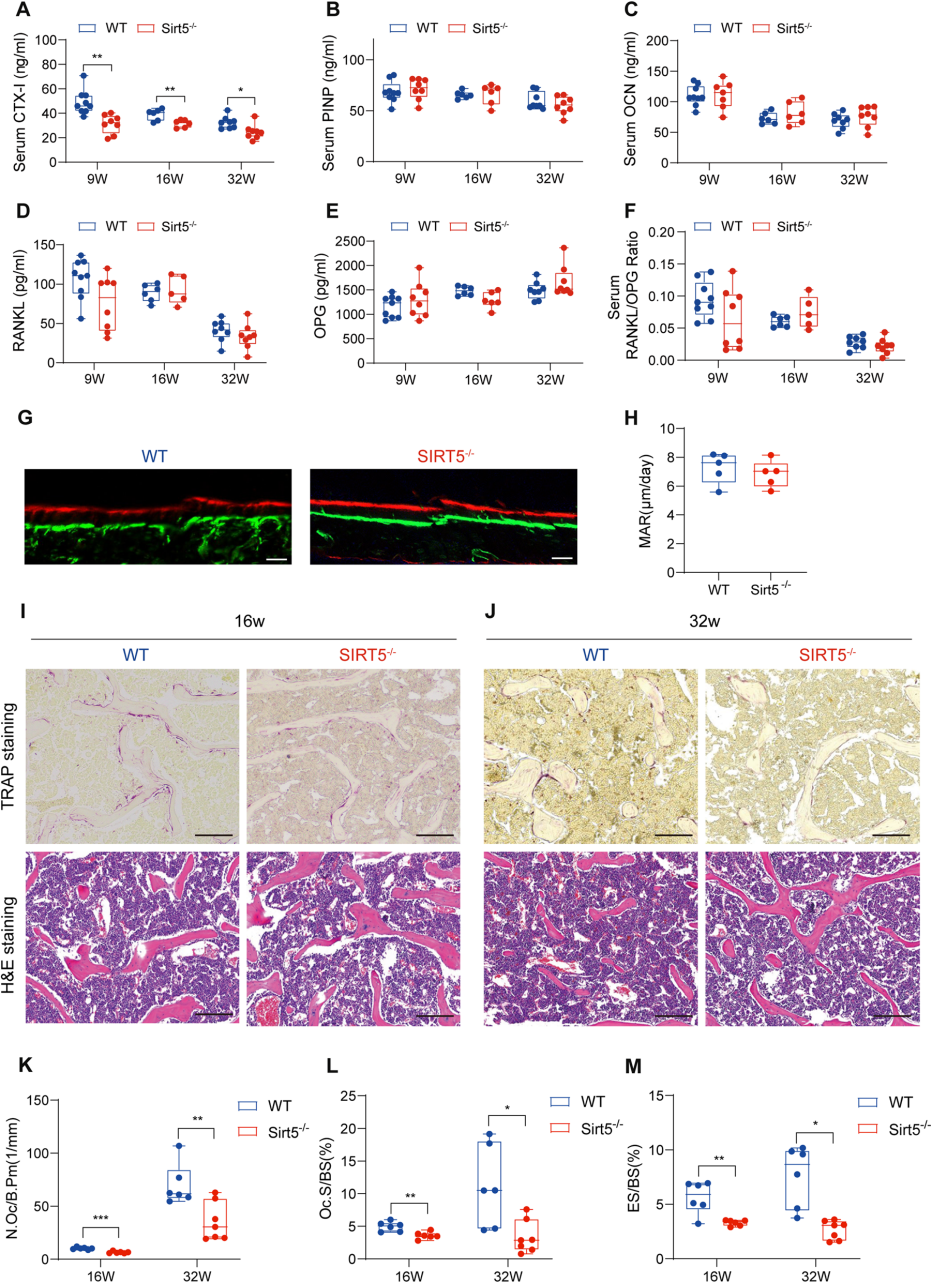
Osteoclast number and activity are reduced in *Sirt5*^−/−^mice. **A**-**F** Serum of WT and *Sirt5*^−/−^ mice at 9, 16 and 32 weeks of age were collected to perform ELISA assays (*n* = 6–9). The levels of serum bone resorption indexes (CTX-I) were detected by ELISA (**A**). The levels of serum bone formation indexes (PINP and OCN) were detected by ELISA (**B**-**C**). Serum RANKL and OPG were detected by ELISA (**D**-**E**). And the ratio of RANKL/OPG was presented (**F**). **G** Representative images of calcein-alizarin red S double labeling of femurs from 8-week-old male WT and *Sirt5*^−/−^ mice. Scale bar, 50 μm. **H** Quantified analysis of MAR in distal femur (*n* = 5). **I**-**J** Representative images of TRAP (Top) and H&E staining (Bottom) of distal femoral epiphyses from 16- and 32-week-old WT and *Sirt5*^−/−^ mice. Scale bar, 200 μm. **K**-**M** The quantification of osteoclast number/bone trabecular perimeter (N.Oc/B.Pm) (*n* = 6–7) (**K**). The quantification of osteoclast surface/bone trabecular surface (Oc.S/BS) (*n* = 6–7) (**L**). The quantification of eroded surface/bone surface (ES/BS) (*n* = 6–7) (**M**). The data are presented as means ± SD. ^*^p < 0.05, ^**^p < 0.01, ^***^p < 0.001 vs. WT

As bone homeostasis relies on the equilibrium between bone formation and bone resorption [20], we performed detailed investigations on whether the improved bone phenotype in *Sirt5*^−/−^ mice was attributed to increased bone formation or reduced bone resorption; specifically, we measured potential changes in the serum levels of bone turnover markers. Compared with WT mice at 9, 16 and 32 weeks of age, *Sirt5*^−/−^ mice presented a decrease in the serum levels of the bone resorption marker C-terminal telopeptide of type I collagen (CTX-I) (Fig. 2A); however, no significant changes in the levels of the bone formation markers N-terminal propeptide of type I procollagen (PINP) and osteocalcin (OCN) were observed (Fig. 2B and C). In addition, there were no significant differences in the serum levels of RANKL or osteoprotegerin (OPG) or in the RANKL/OPG ratio between the two mouse groups (Fig. 2D-F). Calcein-alizarin red S double labeling was performed in *Sirt5*^−/−^ mice and their littermate controls to evaluate bone formation level. As shown in Fig. 2G and H, deletion of *Sirt5* has no effect on cortical mineral apposition rate (MAR) in femurs. Immunohistochemical (IHC) staining for SP7 also revealed no difference in the osteoblast number/bone perimeter (N.Ob/B.Pm) between femur tissue sections from WT mice and those from *Sirt5*^−/−^ mice (Fig. S1).These results suggest that *Sirt5* deletion has no effect on the number or activity of osteoblasts. Instead, increased bone mass in the *Sirt5*^−/−^ mice may be attributed to reduced bone resorption.

To test this hypothesis, we conducted tartrate-resistant acid phosphatase (TRAP) staining and hematoxylin-eosin (HE) staining on femur tissue sections from 16-week-old and 32-week-old mice. There were fewer mature osteoclasts in the *Sirt5*^−/−^ mice than in the WT mice (Fig. 2I and J). Quantitative analysis of TRAP-positive mature osteoclasts revealed a noticeable decrease in the osteoclast number/bone perimeter (N.Oc/B.Pm) (Fig. 2K) and on the osteoclast surface/bone surface (Oc. S/BS) (Fig. 2L). Consistently, the eroded surface/bone surface (ES/BS) ratio is also reduced in *Sirt5*^−/−^ mice (Fig. 2M). These data collectively indicate a reduced quantity and bone resorption area of osteoclasts in *Sirt5*^−/−^ mice.

### SIRT5 stimulates osteoclast differentiation in vitro

**Fig. 3:**
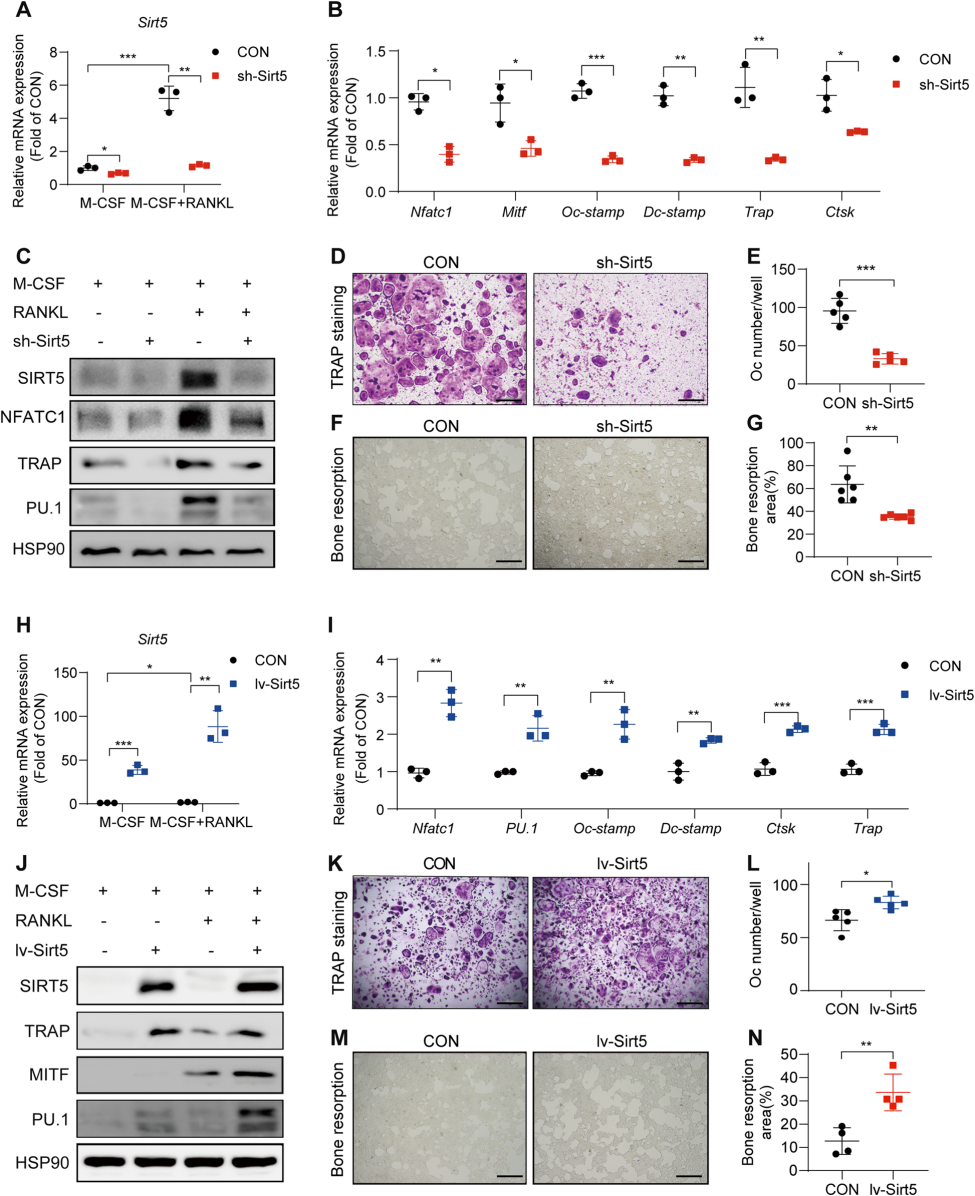
SIRT5 stimulates osteoclast differentiation in vitro.** A** Relative mRNA expression of *Sirt5* in empty lentivirus (CON) and lentivirus-mediated *Sirt5* knockdown (sh-*Sirt5*) BMDMs after 3-day treatment of 20 ng/mL MCSF or 20 ng/mL MCSF + 50 ng/mL RANKL (*n* = 3). **B** Relative mRNA expression of osteoclastogenesis-related genes including early osteoclastic differentiation markers (*Nfatc1* and *Mitf*), osteoclast fusion markers (*Dc-stamp* and *Oc-stamp*), and osteoclast functional markers (*Trap* and *Ctsk*) (*n* = 3). **C** Expression levels of SIRT5 and osteoclastogenesis-related proteins in CON and sh-*Sirt5* BMDMs after 4 days with or without RANKL induction. **D-E** CON and sh-*Sirt5* BMDMs were induced with MCSF and RANKL for 5 days, and then TRAP staining was used to identify TRAP-positive multinucleated cells (**D**). Scale bar, 200 μm. The number of TRAP-positive multinucleated cells was calculated and presented graphically (*n* = 5) (**E**). **F**-**G** The effect of sh-*Sirt5* on the bone resorption of osteoclasts. Images of the resorbed area on synthetic carbonate apatite-coated surfaces 7 days after induction by MCSF and RANKL (**F**). Scale bar, 200 μm. The areas of the resorption pits were quantified and presented graphically (*n* = 6) (**G**). **H** Relative mRNA expression of *Sirt5* in empty lentivirus (CON) and lentivirus-mediated *Sirt5* overexpression (lv-*Sirt5*) BMDMs after 3-day treatment of MCSF or MCSF and RANKL (*n* = 3). **I** Relative mRNA expression of osteoclastogenesis-related genes (*n* = 3). **J** Expression levels of osteoclastogenesis-related proteins in CON and lv-*Sirt5* BMDMs after 4 days with or without RANKL induction (*n* = 3). **K-L** CON and lv-*Sirt5* BMDMs were induced with MCSF and RANKL for 5 days, and then TRAP staining was used to identify TRAP-positive multinucleated cells (**K**). Scale bar, 200 μm. The number of TRAP-positive multinucleated cells was calculated and presented graphically (*n* = 5) (**L**). **M-N** Images of bone resorption areas in the CON and lv-*Sirt5* groups. (**M**). Scale bar, 200 μm. Resorption pit areas were quantified and graphed (*n* = 4) (**N**). The data are presented as means ± SD. ^*^p < 0.05, ^**^p < 0.01, ^***^p < 0.001 vs. CON

**Fig. 4:**
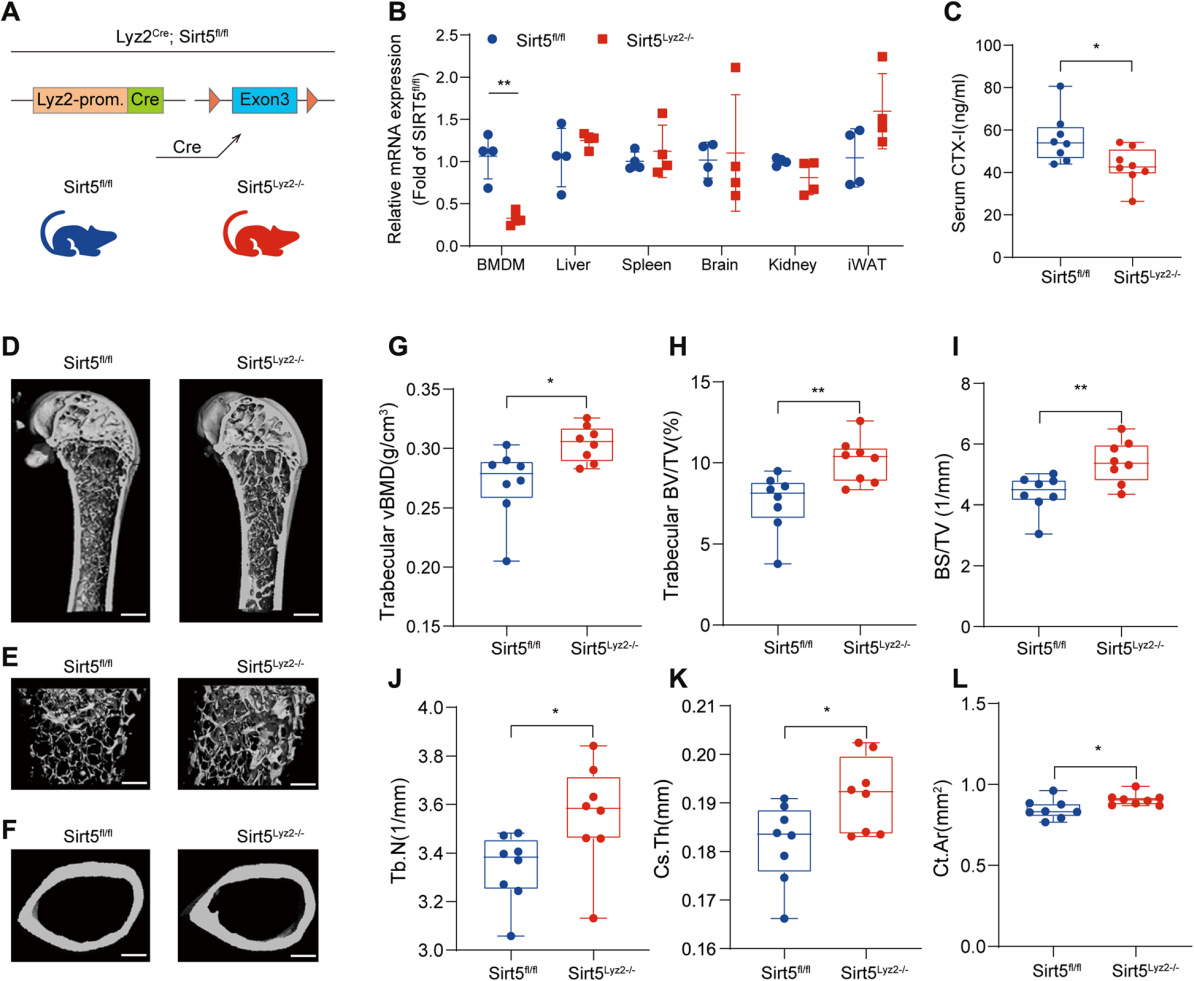
Conditional *Sirt5* deletion in bone marrow increases bone mass in mice. **A** Illustration of *Sirt5* conditional knockout in *Lyz2*-expressing monocytic lineage cells. **B** qRT-PCR analysis of mRNA levels of *Sirt5* in various tissues of *Sirt5*^fl/fl^ and *Sirt5*^*Lyz2*−/−^ mice (*n* = 4). **C** The level of serum CTX-I was detected by ELISA (*n* = 8). **D-F** Representative 3D image reconstruction of coronal sections (**D**, scale bar, 800 μm), trabecular bone (E, scale bar, 400 μm) and cortical bone (F, scale bar, 450 μm) of distal femurs from male *Sirt5*^fl/fl^ and *Sirt5*^*Lyz2*−/−^ mice at 32 weeks of age. **G**-**L** Quantitative analysis of trabecular vBMD (**G**), BV/TV (**H**), surface area density (BS/TV) (**I**) and trabecular number (Tb.N) (**J**), as well as Ct.Th (**K**) and cortical area (Ct.Ar) (**L**) by microCT (*n* = 8). The data are presented as means ± SD. ^*^p < 0.05, ^**^p < 0.01 vs. *Sirt5*^fl/fl^

To further investigate the impact of SIRT5 on osteoclasts, primary BMDMs were isolated from mice and transfected with lentivirus-delivered short hairpin RNA targeting *Sirt5* (sh-*Sirt5*). We subsequently induced the differentiation of BMDMs into osteoclasts with RANKL. *Sirt5* expression increased by 4- to 6-fold following the induction of differentiation (Fig. 4A). The data from a study comparing the gene expression of human osteoclast (OC)-like cells with that of their precursor peripheral blood mononuclear cells (PBMC) [22] indicate that *Sirt5* is upregulated during human osteoclast differentiation (Figure S2). Nuclear factor of activated T-cells, cytoplasmic 1 (*Nfatc1*), microphthalmia-associated transcription factor (*Mitf*), and PU.1 are key transcription factors required for osteoclast commitment and early differentiation [23]. Dendritic cell-specific transmembrane protein (*Dc-stamp*) and osteoclast stimulatory transmembrane protein (*Oc-stamp*) regulate the fusion process during osteoclastogenesis [24, 25]. *Trap* and cathepsin K (*Ctsk*) are two efficient enzymes that are involved in the functions of osteoclasts. As shown in Fig. 3B, the mRNA expression levels of *Nfatc1*, *Mitf*, *Dc-stamp*, *Oc-stamp*, *Trap*, and *Ctsk* were markedly decreased after *Sirt5* was silenced. The protein expression levels of NFATC1, PU.1, and TRAP showed similar trends (Fig. 3C).

Furthermore, the differentiation of *Sirt5*-knockdown osteoclasts was assessed by TRAP staining and a bone resorption assay. The RANKL-induced formation of TRAP-positive multinucleated cells was significantly reduced in the *Sirt5*-knockdown group (Fig. 3D and E). Compared with that in the control group, the area of resorbed pits formed by osteoclasts in the *Sirt5*-knockdown group was smaller (Fig. 3F and G).

To further confirm the regulatory role of SIRT5 in osteoclast differentiation and function, we transfected BMDMs with a *Sirt5*-overexpressing lentivirus (lv-*Sirt5*) (Fig. 3H). After inducing differentiation into osteoclasts, the mRNA expression levels of *Nfatc1*, PU.1, *Oc-stamp*, *Dc-stamp*, *Ctsk*, and *Trap* were significantly increased in the *Sirt5*-overexpressing BMDMs (Fig. 3I). Similar results were observed for the protein expression of TRAP, PU.1, and *Mitf* in BMDMs overexpressing *Sirt5* (Fig. 3J). TRAP staining demonstrated that *Sirt5* overexpression promoted osteoclast differentiation (Fig. 3K and L). The area of resorbing pits formed by the *Sirt5*-overexpressing osteoclasts was larger than that formed by the control osteoclasts (Fig. 3M and N). These results suggest that SIRT5 is essential for osteoclast differentiation.

### Conditional *Sirt5* deletion in the monocytic lineage increases bone mass in mice

To further confirm that the increased bone mass and improved bone microstructure observed in *Sirt5*^−/−^ mice occur due to direct effects of SIRT5 on osteoclast differentiation, we generated *Sirt5*^Lyz2−/−^ mice by crossing *Sirt5*^flox/flox^ mice with Lyz2-cre mice (Fig. 4A). Analysis of *Sirt5* mRNA levels in different tissues of *Sirt5*^Lyz2−/−^ mice verified the specific deletion of *Sirt5* in BMDMs (Fig. 4B). The serum level of the bone resorption marker CTX-I was decreased in the *Sirt5*^Lyz2−/−^ mice (Fig. 4C).

Micro-CT analysis was conducted to evaluate the impact of monocytic lineage-specific *Sirt5* deletion on the bone phenotype of the mice. As shown in Fig. 4D-F and 32-week-old *Sirt5*^Lyz2−/−^ mice presented an increase in the bone mass of distal femurs. Microstructure analysis of the distal femurs revealed improved trabecular vBMD, BV/TV, BS/TV, and Tb.N values, as well as elevated Ct.Th and cortical area (Ct.Ar) values in the *Sirt5*^Lyz2−/−^ mice (Fig. 4G-L). HE staining revealed an increased number of trabeculae in the lumbar vertebrae of the *Sirt5*^Lyz2−/−^ mice (Fig. S3). TRAP staining of histological sections of lumbar vertebrae also demonstrated that the N.Oc/B.Pm and Oc. S/BS values were decreased in *Sirt5*^Lyz2−/−^ mice (Fig. S3). These findings confirm that SIRT5 plays an essential role in osteoclastogenesis.

### Inhibition of SIRT5 activity antagonizes OVX-induced bone loss in mice

**Fig. 5:**
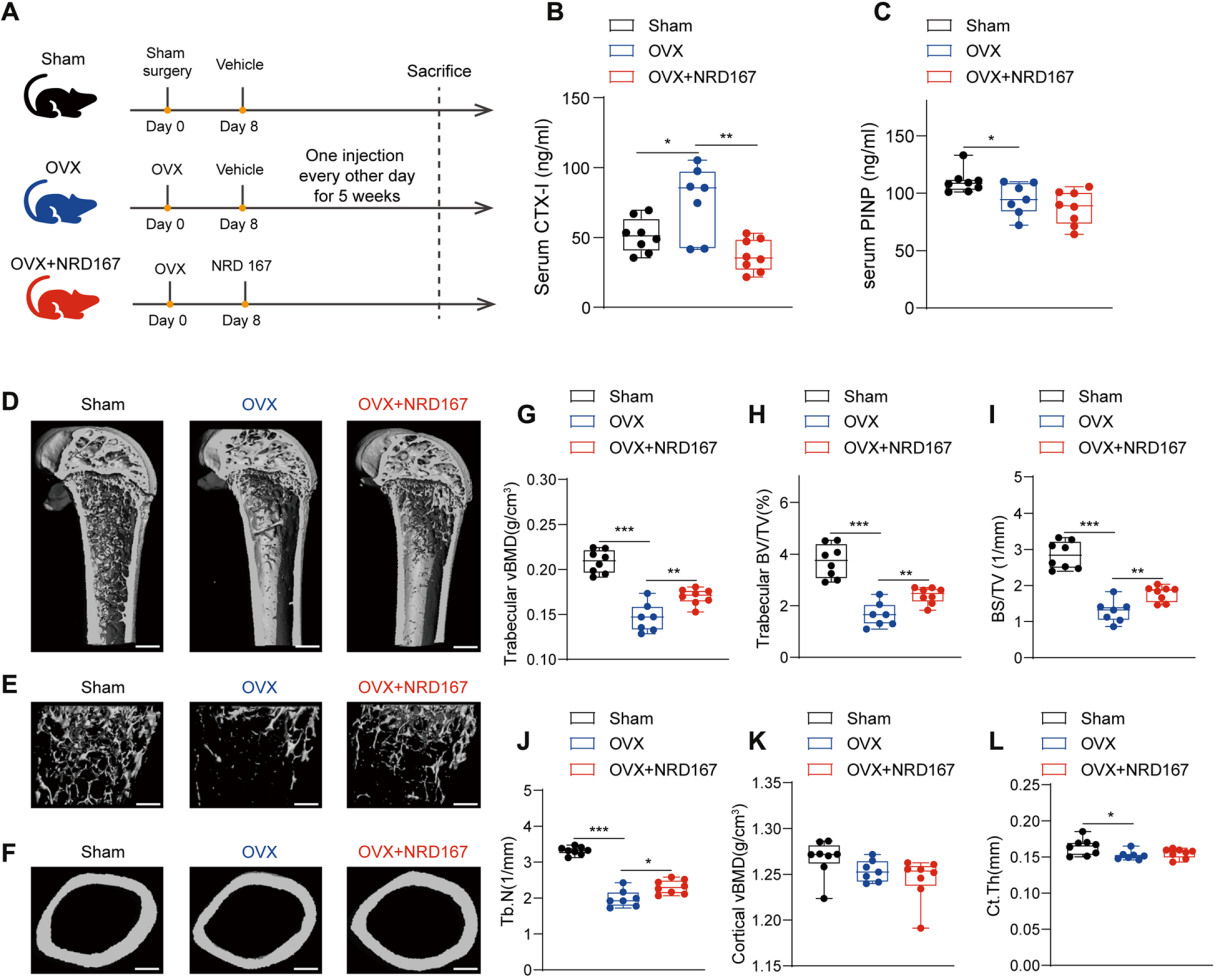
Inhibition of SIRT5 activity rescues OVX-induced bone loss in mice. **A** C57BL/6 mice were randomly assigned to three treatment groups, and ovariectomy or sham surgery was performed at 8 weeks of age. 1 week later, NRD167 or vehicle were administered to mice via intraperitoneal injection every other day, followed by sample collection after 5 weeks. **B**-**C** Serum of sham, OVX and OVX + NRD167 mice at 14 weeks of age were collected to perform ELISA assays. The levels of serum CTX-I (**B**) and PINP (**C**) were detected by ELISA. **D**-**F** Representative 3D image reconstruction of coronal sections (**D**, scale bar, 800 μm), trabecular bone (**E**, scale bar, 400 μm) and cortical bone (**F**, scale bar, 450 μm) of distal femurs from sham, OVX and OVX + NRD167 mice at 14 weeks of age. **G-L** Quantitative analysis of trabecular vBMD (**G**), BV/TV (**H**), surface area density (BS/TV) (**I**) and trabecular number (Tb.N) (**J**), as well as cortical vBMD (K) and Ct.Th (L) by microCT. The data are presented as means ± SD (*n* = 7–8). ^*^p < 0.05, ^**^p < 0.01, ^***^p < 0.001 vs. Sham

As shown in Fig. 5A, female mice were evenly divided into 3 groups (*n* = 8 per group): the sham model (sham) group, the OVX model (OVX) group, the experimental OVX plus injection with NRD167, a potent and selective SIRT5 inhibitor [26], (OVX + NRD167) group. One week after surgery, the mice in the sham and OVX groups were injected with vehicle, and the mice in the OVX + NRD167 groups were injected with NRD167 dissolved in vehicle every other day. NRD167 treatment for 5 weeks significantly decreased the serum level of CTX-I in OVX model mice (Fig. 5B), indicating a reduction in bone resorption. However, no significant change in the level of the bone formation marker PINP was observed (Fig. 5C). Micro-CT 3D reconstruction images revealed a substantial decrease in trabecular bone mass (Fig. 5D and E) and a slight reduction in cortical bone thickness (Fig. 5F) in OVX model mice compared with those in sham-operated mice. After NRD167 treatment, the trabecular bone mass was improved in the OVX model mice (Fig. 5D and E), whereas the cortical bone thickness did not significantly change (Fig. 5F). Further analysis of bone microstructure parameters revealed significant increases in the trabecular vBMD, BV/TV, BS/TV, and Tb.N values in the NRD167-treated OVX model mice but no significant improvement in the cortical bone microstructure parameters (Fig. 5G-L, Table S4). These results indicate that the inhibition of SIRT5 activity leads to increased bone mass and improved trabecular bone microstructure in mice with OVX-induced osteoporosis.

### SIRT5 enhances mitochondrial energy production in osteoclasts

**Fig. 6:**
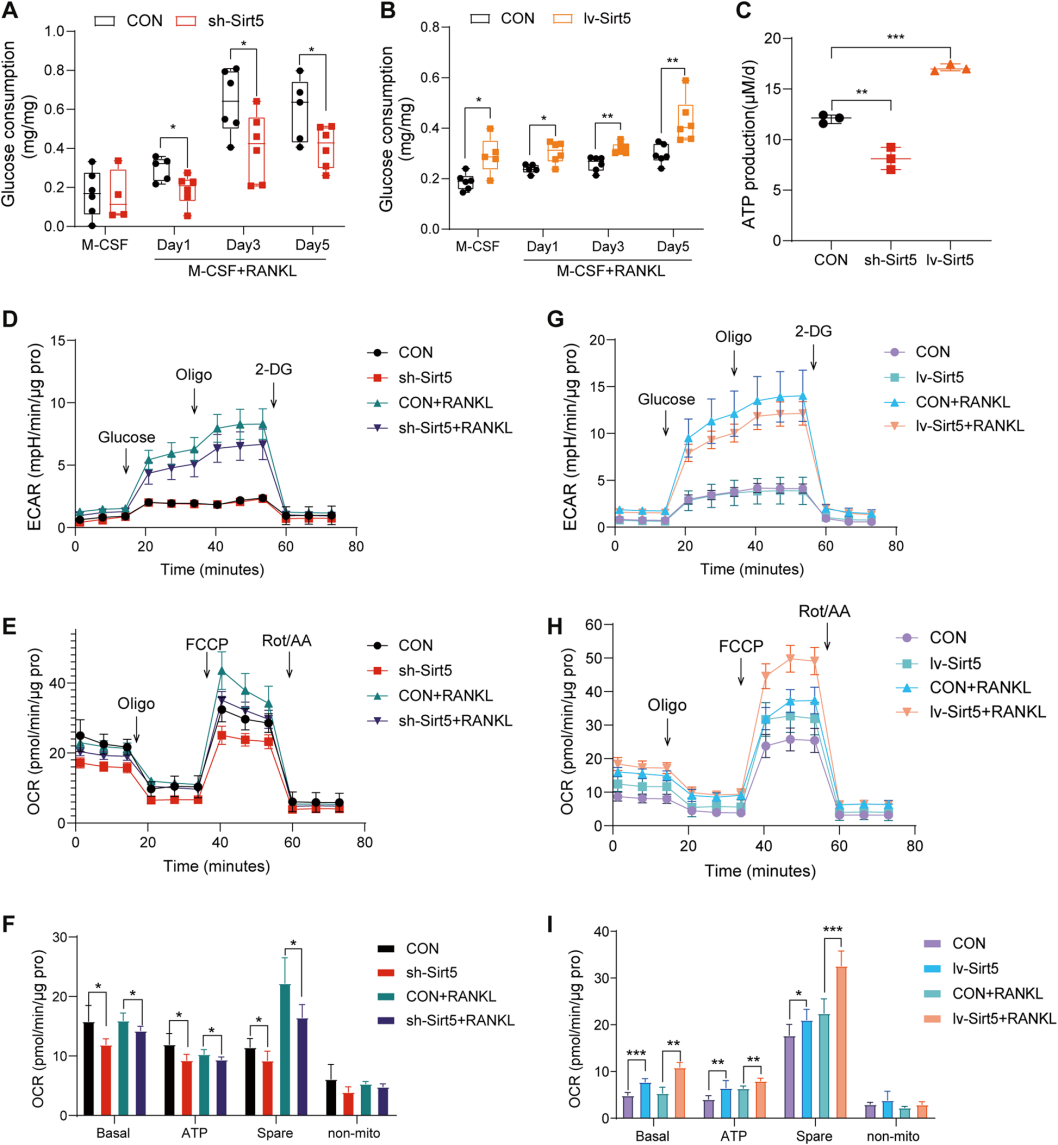
SIRT5 promotes mitochondrial energy production in osteoclasts. **A**-**B** Glucose consumption (normalized to total protein) of control and sh-*Sirt5*(**A**) or lv-*Sirt5*(**B**) BMDMs treated with MCSF and RANKL for 1,3 or 5 days (*n* = 6). **C** After 2 days of MCSF and RANKL treatment, BMDMs were plated into six-well plates at a density of 6 × 10^5^ cells per well. After 24 h, the ATP concentration in the cell lysate was measured (*n* = 3). **D**-**F** Control and sh-*Sirt5* BMDMs were treated with MCSF alone or with both MCSF and RANKL for 2 days, and then seeded onto 96-well Seahorse cell culture plates at a density of 3 × 10^4^ cells per well for glucose glycolysis stress test(**D**) and mitochondrial stress test(**E**). Quantitative statistics of oxygen consumption rate (OCR) at various stages of the mitochondrial stress test(**F**). Results were normalized to the protein levels per well (*n* = 6). **G**-**I** Control and lv-*Sirt5* BMDMs were treated with MCSF alone or with both MCSF and RANKL for 2 days, and then plated onto 96-well Seahorse cell culture plates at a density of 3 × 10^4^ cells per well for glucose glycolysis stress test(**G**) and mitochondrial stress test(**H**). Quantitative statistics of OCR at various stages of the mitochondrial stress test(**I**). Results were normalized to the protein levels per well (*n* = 6). The data are presented as means ± SD. ^*^p < 0.05, ^**^p < 0.01, ^***^p < 0.001 vs. CON or CON + RANKL

SIRT5 is recognized as a mitochondrial matrix protein that regulates the activity of mitochondrial energy metabolism [27]. To further investigate the role of SIRT5 in the mitochondrial metabolism of osteoclasts, we assessed the metabolic capacity of *Sirt5*-knockdown BMDMs. First, BMDMs were transfected with sh-*Sirt5* lentivirus in vitro and treated with MCSF alone or with both MCSF and RANKL for 1, 3, or 5 days. Glucose consumption was measured within 24 h, and the results revealed a reduction in glucose consumption in sh-*Sirt5* BMDMs relative to that in the control group (Fig. 6A); however, lv-Sirt5 BMDMs exhibited elevated glucose consumption compared with that in the control group (Fig. 6B). After 2 days of treatment with MCSF and RANKL, *Sirt5*-knockdown cells exhibited reduced ATP production, whereas *Sirt5*-overexpressing cells exhibited showed the opposite effect (Fig. 6C). To further investigate the reason for the decreased ATP production in BMDMs, glucose glycolysis stress tests and mitochondrial stress tests were conducted with Seahorse assays. *Sirt5* knockdown led to weakened mitochondrial basal metabolism, ATP generation, and spare respiration capacity (Fig. 6E-F), whereas *Sirt5* overexpression had opposite effects (Fig. 6H-I). On the other hand, the glycolytic capacity remained unchanged (Fig. 6D and G). Therefore, SIRT5 primarily promotes mitochondrial metabolism, which is essential for supplying energy in the process of osteoclast differentiation.

### Identification of JIP4 as a target protein of SIRT5

**Fig. 7:**
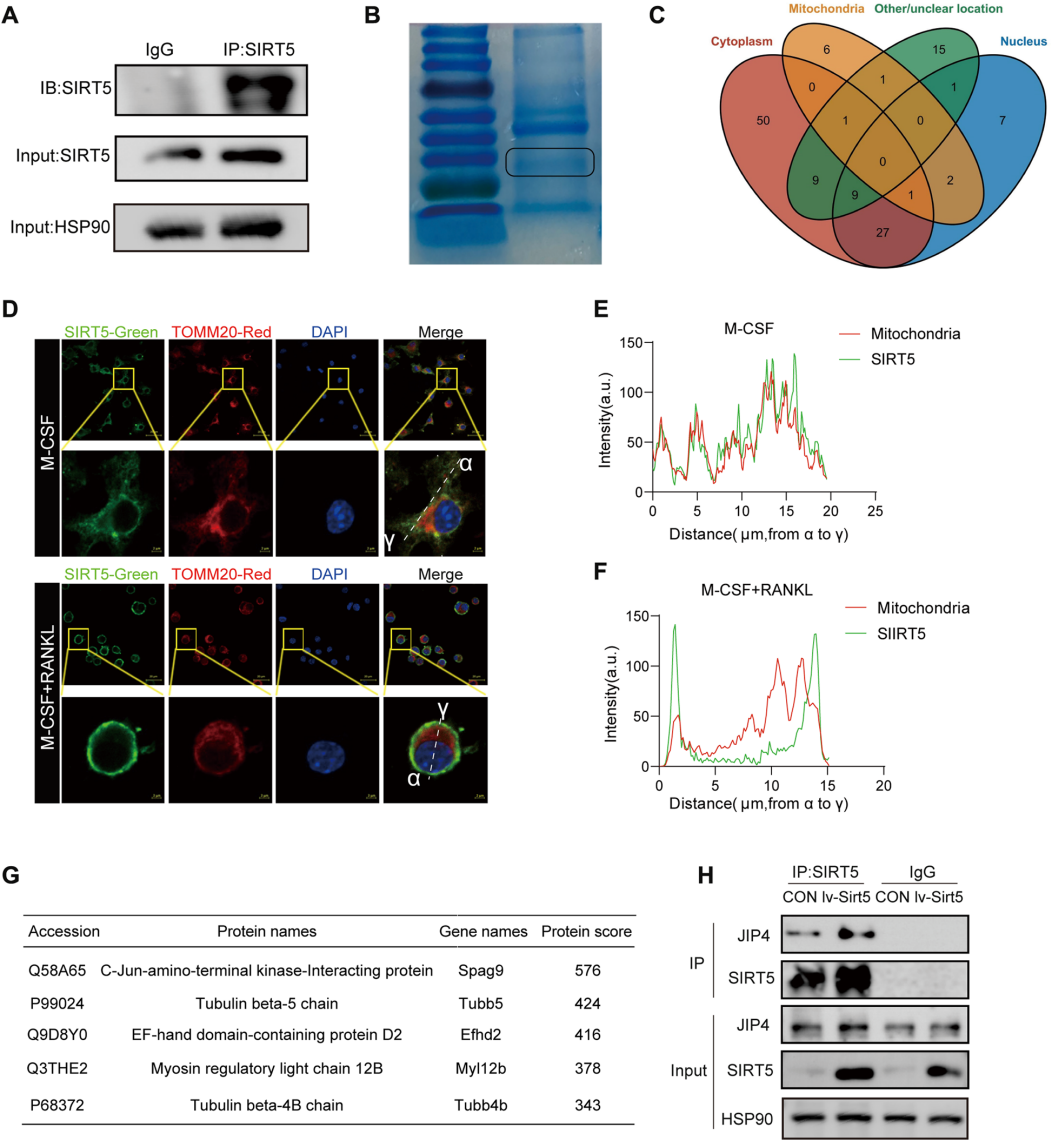
Identification of JIP4 as a target protein of SIRT5. **A**-**B** Protein samples from BMDMs treated with RANKL and M-CSF for 2 days were collected, and SIRT5 was immunoprecipitated (IP-SIRT5) for subsequent Western blot verification (**A**). The gel strip of the IP-SIRT5 sample was subjected to Coomassie Brilliant Blue staining, and the band between 25 kD and 35 kD, indicated by black boxes, represented the band of SIRT5 (**B**). The entire gel strip was further subjected to protein mass spectrometry analysis. **C** After excluding non-specifically bound proteins detected in the IgG negative control, subcellular localization of proteins detected in the IP-SIRT5 mass spectrometry was statistically analyzed and presented using a Venn diagram. The results show the quantity of proteins localized in the cytoplasm, mitochondria, nucleus, and other/unknown locations. **D** Representative immunofluorescence image of SIRT5 (green), the mitochondrial marker TOMM20 (red) and DAPI (blue) in BMDMs treated with MCSF or MCSF and RANKL for 1 day (scale bars, 20 μm/ 2 μm). **E**-**F** The line charts represent the fluorescence intensity of SIRT5 and TOMM20, presenting the distance from α to γ in BMDMs treated with MCSF (**E**) or MCSF and RANKL (**F**). **G** The top five cytoplasmic proteins ranked by protein scores in the IP-MS results were listed. **H** BMDMs were transfected with *Sirt5*-overexpressing lentivirus, treated with MCSF and RANKL for 2 days, and the protein levels of JIP4 and SIRT5 in the IP-SIRT5 products were detected

SIRT5 usually interacts with target proteins to exert its effects [28]. To identify the target proteins of SIRT5 in osteoclast differentiation, we used coimmunoprecipitation (Co-IP) to enrich SIRT5 and its interacting proteins in RANKL-induced preosteoclasts. After confirming the successful IP of SIRT5 by Western blotting analysis (Fig. 7A), the protein bands on the gel were subjected to Coomassie Brilliant Blue staining (Fig. 7B), followed by protein mass spectrometry (MS) analysis. After excluding nonspecifically bound proteins detected in the IgG negative control, we identified a total of 126 proteins that interacted with SIRT5 from the IP-MS results (Supplementary Material 2). Among them, 96 were located in the cytoplasm, 10 in the mitochondria, and 46 in the nucleus (Fig. 7C). This observation highlights that more SIRT5-interacting proteins are localized outside the mitochondria than within the mitochondria, which is consistent with the findings of a previous study [28].

To determine whether SIRT5 is located primarily within the mitochondria or in the cytoplasm during osteoclast differentiation, we assessed the subcellular localization of SIRT5 in both undifferentiated and RANKL-induced preosteoclasts with a cellular immunofluorescence assay. As shown in Fig. 7D, SIRT5 was localized mainly in the mitochondrial region of undifferentiated BMDMs. However, in RANKL-induced preosteoclasts, SIRT5 was localized partially in the cytoplasm. We subsequently examined the intensity-location relationship between the SIRT5 signal and the signal of the mitochondrial marker TOMM20 in the cells. The results revealed that the locations of SIRT5 and the mitochondrial exhibited higher consistency in undifferentiated BMDMs compared to RANKL-induced preosteoclasts (Fig. 7E and F). These findings suggested that in undifferentiated BMDMs, SIRT5 was predominantly localized to mitochondria, whereas in RANKL-induced preosteoclasts, SIRT5 was observed to be localized to both cytoplasm and mitochondria. These results indicate that cytoplasmic SIRT5 may play an important role in osteoclast differentiation.

Among the top 5 cytoplasmic proteins, c-Jun amino-terminal kinase-interacting protein 4 (JIP4, a splice variant of the *Spag9* gene) had the highest score (Fig. 7G). As shown in Fig. S4, the expression of SIRT5 and JIP4 increased at both the gene and protein levels during osteoclast differentiation. This finding indicates the potential involvement of SIRT5 and JIP4 in the process of osteoclast differentiation. Subsequent IP and Western blotting experiments confirmed the interaction between SIRT5 and JIP4 (Fig. 7H). JIP4 is a scaffold protein that can mediate signal transduction by MAPK signaling [29]. In the process of osteoclast differentiation, MAPK signaling plays an indispensable role [30]. Therefore, we explored whether JIP4 is involved in SIRT5-regulated osteoclast differentiation.

### SIRT5 promotes p38 and JNK phosphorylation through interactions with JIP4

**Fig. 8:**
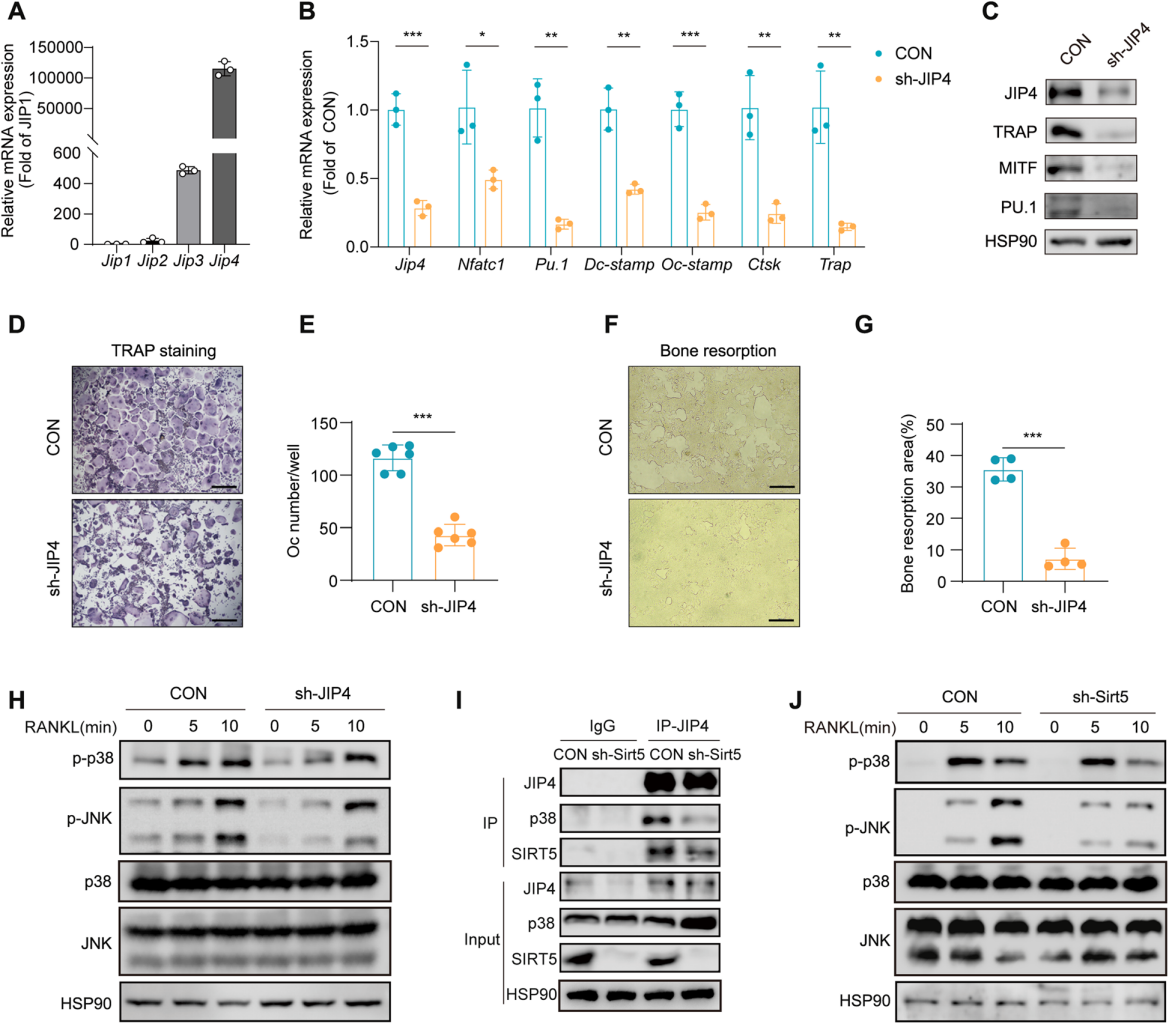
SIRT5 promotes the phosphorylation of p38 and JNK through its interaction with JIP4.** A** Relative mRNA expression of JIP1, JIP2, JIP3, JIP4 in BMDMs after 2-day treatment of MCSF and RANKL (*n* = 3). **B** Relative mRNA expression of JIP4 and osteoclastogenesis-related genes including early osteoclastic differentiation markers (*Nfatc1* and PU.1), osteoclast fusion markers (*Dc-stamp* and *Oc-stamp*), and osteoclast functional markers (*Trap* and *Ctsk*) (*n* = 3). **C** Expression levels of JIP4 and osteoclastogenesis-related proteins in CON, sh-JIP4 BMDMs after 4 days RANKL induction. **D**-**E** CON, sh-JIP4 BMDMs were induced with MCSF and RANKL for 5 days, and then TRAP staining was used to identify TRAP-positive multinucleated cells (**D**). Scale bar, 200 μm. The number of TRAP-positive multinucleated cells was calculated and presented graphically (*n* = 6) (**E**). **F**-**G** Images showing bone resorption areas in the CON and sh-JIP4 groups (**F**). Scale bar, 200 μm. The resorption pit areas were quantified. (*n* = 4) (**G**). **H** After exposing CON and sh-JIP4 BMDMs to RANKL for 0, 5 and 10 min, the phosphorylation levels of p38 and JNK were detected by Western blot. **I** After treating CON and sh-*Sirt5* BMDMs with MCSF and RANKL for 2 days, protein levels of JIP4, p38, and SIRT5 in the IP-JIP4 products of BMDMs were examined. **J** After exposing CON and sh-*Sirt5* BMDMs to RANKL for 0, 5 and 10 min, the phosphorylation levels of p38 and JNK were detected by Western blot. The data are presented as means ± SD. ^*^p < 0.05, ^**^p < 0.01 vs. CON, ^***^p < 0.001 vs. CON + RANKL

JIP4 belongs to the JNK-interacting protein (JIP) family, which contains multiple protein interaction motifs. Each JIP protein can selectively mediate the activation of various downstream signaling factors, such as p38, JNK, and ERK. These proteins are crucial for maintaining a high degree of specificity in intracellular signaling pathways [31]. We examined the gene expression of four JIP family members, namely, JIP1-4, and found that JIP4 presented the highest expression in BMDMs (Fig. 8A). We transfected BMDMs with lentivirus-delivered short hairpin RNA targeting JIP4 (sh-JIP4), which resulted in satisfactory knockdown efficiency of *Jip4* and decreased the mRNA expression of *Nfatc1*, PU.1, *Oc-stamp*, *Dc-stamp*, *Ctsk*, and *Trap* (Fig. 8B). Consistently, the levels of early osteoclast differentiation-related proteins, such as TRAP, MITF, and PU.1, were also decreased (Fig. 8C). TRAP staining of RANKL-induced osteoclasts revealed a reduction in the number of mature osteoclasts after JIP4 knockdown (Fig. 8D and E). The area of resorbed pits formed by osteoclasts in the JIP4-knockdown group was smaller than that in the control group (Fig. 8F and G). These results indicate the involvement of JIP4 in the regulation of osteoclast differentiation.

Given that JIP4 and SIRT5 interact, we further explored whether SIRT5 affects the function of JIP4. The knockdown of JIP4 inhibited the RANKL-induced phosphorylation of p38 and JNK, indicating that JIP4 functions as a scaffold protein involved in the phosphorylation of p38 and JNK in BMDMs (Fig. 8H). Since JIP4 interacts with JNK and p38 to activate specific signaling pathways [32], we investigated whether SIRT5 affects the interaction of JIP4 with p38 and JNK. As shown in Fig. 8I, *Sirt5* knockdown weakened the binding of JIP4 and p38 in RANKL-induced preosteoclasts. Furthermore, *Sirt5* knockdown inhibited the phosphorylation of p38 and JNK in BMDMs after RANKL stimulation (Fig. 8J). These findings suggest that SIRT5 activates p38 and JNK signaling by interacting with JIP4.

## Discussion

During osteoclast differentiation, RANKL stimulation drives the commitment of osteoclast precursors to the multinucleated mature osteoclast lineage, along with increased mitochondrial biogenesis and increases in mitochondrial size and quantity [17, 33]. Furthermore, the MAPK-mediated activation of downstream gene transcription is indispensable for osteoclast differentiation after RANKL stimulation [34]. In this study, we observed significant induction of the NAD^+^-dependent deacetylase SIRT5 in response to RANKL stimulation, which subsequently led to increased osteoclast differentiation by interacting with JIP4, a scaffold protein abundant in osteoclasts, and increasing the MAPK signaling cascade and mitochondrial energy production. Additionally, both global and conditional monocytic lineage *Sirt5* knockout mice exhibited increased bone mass and strength due to robust decreases in the numbers and functions of osteoclasts. Taken together, our findings highlight the critical role of the SIRT5-JIP4 axis in regulating osteoclast differentiation.

SIRT3-5 are traditionally considered to be predominantly localized in the mitochondria, where they regulate the acylation of various metabolic enzymes to affect their activities [35]. However, a recent study revealed that SIRT3 also localizes to the cell nucleus to regulate histone deacetylation [36]. The subcellular localization of SIRT5 has been a subject of controversy. There are two isoforms of SIRT5 (SIRT5iso1 and SIRT5iso2). In both human and murine systems, SIRT5iso1 is the predominant form, and it is localized to both the mitochondria and the cytoplasm [37]. In addition, a study identified cytoplasmic glycolytic enzymes as targets of SIRT5 through succinylation mass spectrometry [38]. Consistent with a previous finding that more SIRT5-interacting proteins localize outside the mitochondria than SIRT3 and SIRT4 do [39], we found through IP-mass spectrometry that the proteins that interact with SIRT5 in osteoclasts are located predominantly outside the mitochondria. Our study revealed the upregulation of SIRT5 during osteoclast differentiation. Furthermore, cellular immunofluorescence assays indicated that the increased SIRT5 was partially localized to the cytoplasm. The present study reveals for the first time the subcellular localization of SIRT5 during osteoclastogenesis. These findings provide inspiration for identifying target proteins of SIRT5 in the cytoplasm and investigating its extramitochondrial functions.

MAPKs serve as pivotal mediators of the transduction of extracellular signals into intracellular signals, intricately regulating diverse cellular activities, such as gene expression, cell cycle progression, metabolism, motility, survival, apoptosis, and differentiation [40]. Upon RANKL stimulation of osteoclast precursors, the interaction of RANKL with the extracellular domain of RANK triggers the assembly of a RANK homotrimer. This event facilitates the recruitment of TRAF adaptor proteins, particularly TRAF6, to the cytoplasmic tail of the receptor, leading to the activation of downstream MAPK signaling cascades [5]. In the present study, we showed that the expression level of SIRT5 was elevated after RANKL stimulation during osteoclast differentiation. Knockdown of *Sirt5* in bone marrow-derived precursor cells (BMMs) resulted in a significant decrease in the phosphorylation of p38 and JNK, two major MAPK proteins, which consequently led to impaired osteoclastogenesis. Conversely, the overexpression of *Sirt5* had the opposite effect. These results suggest that SIRT5 may participate in osteoclastogenesis by regulating MAPK signaling cascades.

Recently, a number of scaffold proteins, which bring together and organize components of a signaling cascade to facilitate MAPK activation, have been demonstrated to play crucial roles in the regulation of the MAPK signaling network [11]. In this study, we identified a specific JNK-interacting protein called JIP4, which is encoded by the Spag9 gene [31], through IP-mass spectrometry. JIP4 serves as a scaffold protein that facilitates the assembly of MAPKKKs, MAPKKs, JNK, and p38, leading to the activation of both the JNK and p38 MAPK signaling pathways [32]. A study demonstrated that JIP4 coordinates CD40-dependent p38 and JNK signal transduction during B lymphocyte activation [41]. Furthermore, JIP4 has been reported to play a role in transducing tumor necrosis factor (TNF) receptor signals into intracellular signals during the activation of T cells and B cells [41, 42]. Our results showed that the knockdown of JIP4 led to the inhibition of RANKL-induced p38 and JNK phosphorylation, indicating that JIP4 functions as a scaffold protein essential for MAPK signal transduction during osteoclast differentiation. Additionally, the deletion of SIRT5 in osteoclasts significantly impaired the interaction between JIP4 and p38/JNK. Based on these findings, we propose a novel molecular mechanism by which SIRT5 interacts with JIP4 to finely regulate p38 and JNK signal transduction during osteoclast differentiation. Regarding the mechanisms by which SIRT5 regulates JIP4, it is conceivable that SIRT5 might activate JIP4 by deacylation. However, we failed to find any acylation sites through IP-MS or an acylated JIP4 band through IP-WB. In addition to deacylation, another hypothesis is that SIRT5 performs an allosteric regulatory function, enhancing the stability of the JIP4-MAPK complex and thereby promoting downstream signal transduction. A previous study reported that to sense extracellular hypertonicity or osmotic pressure, the guanine nucleotide exchange factor BRX activates downstream MAPK signaling through direct interaction with JIP4 in immune cells [43]. Similarly, our study revealed that SIRT5 promotes MAPK signaling activation by binding to JIP4 in response to RANKL stimulation. These results suggest that the role of SIRT5 in osteoclasts is similar to that of BRX in immune cells, as SIRT5 senses RANKL signals during osteoclastogenesis. A previous study suggested that allosteric inputs from different effectors participate in the regulation of a protein’s activity through functional cross-talk. For example, when oxalate is present, PKM2 adopts an active tetrameric conformation that favors the binding of serine over cysteine [21]. This evidence suggests that BRX and SIRT5 may act as allosteric activators in altering the protein conformation of JIP4, thereby regulating its function. The specific mechanism underlying the regulation of JIP4 protein conformation awaits further elucidation.

SIRT5 modulates the activity of diverse metabolic enzymes that are involved in energy pathways through posttranslational protein modifications such as deacetylation, desuccinylation, deglutarylation, and demalonylation [27]. Studies have shown that SIRT5 facilitates the desuccinylation and activation of isocitrate dehydrogenase 2 (IDH2) and oxoglutarate dehydrogenase (OGDH), thereby promoting the tricarboxylic acid cycle [44, 45]. In the present study, we revealed that SIRT5 may regulate osteoclastogenesis by enhancing mitochondrial respiration during osteoclast differentiation while leaving the glycolytic capacity unchanged. Mechanistically, we identified an interaction between OGDH and SIRT5 in osteoclasts by IP-MS (Table S7), suggesting that SIRT5 potentially activates OGDH via desuccinylation, as previously reported [45]. Together, these findings highlight the potential role of SIRT5 as a key orchestrator of mitochondrial metabolism in regulating osteoclastogenesis.

As reported, some mitochondrial protein precursors are synthesized in the cytoplasm [46]. They enter the mitochondria in an unfolded state, where they are subsequently cleaved and folded into mature proteins [47]. This process is typically unidirectional. The present study found that the cytoplasmic SIRT5 increased during osteoclast differentiation. However, this does not suggest that the elevated SIRT5 is translocated from the mitochondria; it is also likely to be derived from newly synthesized SIRT5 during osteoclast differentiation. The data in Fig. 6 suggest that SIRT5 can affect mitochondrial function in both undifferentiated BMDMs and RANKL-induced preosteoclasts. Elevation of cytoplasmic SIRT5 did not result in losing SIRT5 regulation of mitochondrial function. Whether the elevated cytoplasmic SIRT5 is translocated from the mitochondria or synthesized in the cytoplasm remains to be investigated in the future.

The present study is limited by the absence of data about the relationship between SIRT5 mutations and changes in bone mass in the population. Further research from mice studies to human investigations is therefore needed [48]. Furthermore, the precise molecular mechanisms underlying the interaction between SIRT5 and JIP4 at the structural level require elucidation through future research.

In conclusion, we identified a previously unknown function of SIRT5 as a regulator of osteoclast differentiation. Mechanistically, this study reveals that the SIRT5-JIP4 axis is a novel positive regulator that finely orchestrates RANKL-induced osteoclast differentiation and suggests that targeting this axis is a therapeutic strategy for treating osteoporotic bone loss.

## Supplementary Information

Supplementary Material 1.

Supplementary Material 2.

## Data Availability

No datasets were generated or analysed during the current study.
