# Supplementary material for: The SIRT5-JIP4 interaction promotes osteoclastogenesis by modulating RANKL-induced signaling transduction
Source: Cell Commun Signal. 2025 Jan 14;23:26. doi: 10.1186/s12964-024-02021-x (PMC11730813; doi:10.1186/s12964-024-02021-x)
Supplement: Supplementary file 1 — Supplementary Material 1. [file 12964_2024_2021_MOESM1_ESM.docx]

Table.S1

Quantitative analysis of distal femurs from WT and SIRT5-KO mice by Micro-CT

Parameter WT Sirt5^-/-^  *p* value

**Trabecular parameters, mean ± SD**

vBMD (g/cm^3^) 0.184 ± 0.021 0.240 ± 0.038 0.009482

BV/TV (%) 9.363 ± 1.676 15.114 ± 1.795 0.013594

BS/TV (1/mm) 6.906 ± 1.318 10.067 ± 1.974 0.008533

Tb.Th (mm) 0.052 ± 0.002 0.055 ± 0.004 0.209466

Tb.N (1/mm) 1.785 ± 0.396 2.715 ± 0.593 0.009599

Tb.Sp (mm) 0.254 ± 0.017 0.210 ± 0.018 0.001320

SMI 2.161 ± 0.220 1.824 ± 0.244 0.031039

Tb.Pf(1/mm) 26.967 ± 4.368 20.937 ± 4.978 0.049795

**Cortical parameters, mean ± SD**

vBMD (g/cm^3^) 0.968 ± 0.012 0.985 ± 0.011 0.038265

BV/TV (%) 79.050 ± 0.893 80.575 ± 1.125 0.026996

Ct.Th (mm) 0.217 ± 0.012 0.233 ± 0.011 0.037462

Table.S2

Quantitative analysis of distal femurs from WT and *Sirt5* KO mice by Micro-CT

Parameter WT Sirt5^-/-^ *p* value

**Trabecular parameters, mean ± SD**

vBMD (g/cm^3^) 0.129 ± 0.020 0.228 ± 0.053 0.000472

BV/TV (%) 3.745 ±1.810 13.463 ± 5.735 0.000891

BS/TV (1/mm) 2.804 ±1.269 8.311 ± 2.912 0.000481

Tb.Th (mm) 0.055 ± 0.004 0.058 ± 0.003 0.113238

Tb.N (1/mm) 0.685 ± 0.356 2.284 ± 0.915 0.000819

Tb.Sp (mm) 0.355 ± 0.051 0.244 ±0.035 0.000255

SMI 2.721 ± 0.302 1.868 ± 0.502 0.001807

Tb.Pf(1/mm) 34.507 ± 5.011 20.394 ± 7.601 0.001093

**Cortical parameters, mean ± SD**

vBMD (g/cm^3^) 0.966 ± 0.032 0.990 ± 0.020 0.103415

BV/TV (%) 78.223 ± 1.250 80.502 ± 1.645 0.010559

Ct.Th (mm) 0.211 ± 0.015 0.236 ± 0.020 0.014566

Table.S3

Quantitative analysis of distal femurs from Sirt5^fl/fl^ and Sirt5^Lyz2-/-^ mice by Micro-CT

Parameter Sirt5^fl/fl^  Sirt5^Lyz2-/-^  *p* value

**Trabecular parameters, mean ± SD**

vBMD (g/cm^3^) 0.271 ± 0.030 0.304 ± 0.015 0.014650

BV/TV (%) 7.576 ± 1.816 10.153 ± 1.387 0.006532

BS/TV (1/mm) 4.379 ± 0.625 5.413 ± 0.710 0.00793

Tb.Th (mm) 0.045 ± 0.005 0.049 ± 0.004 0.209466

Tb.N (1/mm) 3.337 ± 0.141 3.554 ± 0.215 0.031482

Tb.Sp (mm) 0.295 ± 0.014 0.277 ± 0.017 0.034275

SMI 2.369 ± 0.384 1.950 ± 0.278 0.025416

Conn.D(1/mm^3^) 51.374 ± 8.078 71.775 ±21.152 0.023183

**Cortical parameters, mean ± SD**

vBMD (g/cm^3^) 1.353 ± 0.018 1.356 ± 0.026 0.809970

Ct.Ar (mm^2^) 0.847 ± 0.061 0.907 ± 0.038 0.032001

Ct.Th (mm) 0.182 ± 0.008 0.192 ± 0.008 0.025661

Table.S4

Quantitative analysis of distal femurs from OVX and OVX+NRD167 mice by Micro-CT

Parameter Sirt5^fl/fl^  Sirt5^Lyz2-/-^  *p* value

**Trabecular parameters, mean ± SD**

vBMD (g/cm^3^) 0.146 ± 0.016 0.170 ± 0.009 0.003176

BV/TV (%) 1.645 ± 0.472 2.402 ± 0.317 0.002719

BS/TV (1/mm) 1.285 ± 0.315 1.773 ± 0.213 0.003498

Tb.Th (mm) 0.037 ± 0.004 0.039 ± 0.002 0.094339

Tb.N (1/mm) 1.990 ± 0.240 2.309 ± 0.192 0.013566

Tb.Sp (mm) 0.512 ± 0.058 0.440 ± 0.037 0.012117

SMI 3.097 ± 0.127 3.031 ± 0.155 0.392406

Conn.D(1/mm^3^) 15.343 ± 4.665 21.511 ±7.021 0.070474

**Cortical parameters, mean ± SD**

vBMD (g/cm^3^) 1.254 ± 0.011 1.245 ± 0.023 0.305959

Ct.Ar (mm^2^) 0.657 ± 0.047 0.661 ± 0.031 0.829167

Ct.Th (mm) 0.157 ± 0.014 0.155 ± 0.007 0.767982

Table S5

| Gene Name | Primer Sequence (Forward: 5’ -3’) | Primer Sequence (Reverse: 5’ -3’) |
| --- | --- | --- |
| Rn18s | GCAATTATTCCCCATGAACG | GGCCTCACTAAACCATCCAA |
| Sirt5 | TGATGCGACCTCTCCTGATTGC | TTTCTCCAGTAACCTCCAGCGC |
| Nfatc1 | TGGGAGATGGAAGCAAAGACTGA | CATTGGCAGGAAGGTACGTGAA |
| PU.1 | AGGAGTCTTCTACGACCTGGA | GAAGGCTTCATAGGGAGCGAT |
| Mitf | CAAATGGCAAATACGTTACCCG | CTCCCTTTTTATGTTGGGAAGGT |
| Oc-stamp | CTGTAACGAACTACTGACCCAGC | CCCAGGCTTAGGAAGACGAAG |
| Dc-stamp | GGGGACTTATGTGTTTCCACG | ACAAAGCAACAGACTCCCAAAT |
| Trap | CACTCCCACCCTGAGATTTGT | CATCGTCTGCACGGTTCTG |
| Ctsk | CGCTCACAGTAGCCACGCTT | CCGAGAGATTTCATCCACCTTGCT |
| Alp | CCAGGGGTACAAGGCTAGATGG | AGTTCAGTGCGGTTCCAGACAT |
| Osx | AAGGTAGTGAACAGACTCCGGC | CTCGTCACAAGCAGGGTTAAGC |
| Col1a1 | TCCTGCCGATGTCGCTATC | CAAGTTCCGGTGTGACTCGT |
| Osx | CAAGTTCCGGTGTGACTCGT | ACCTTCCTCTACCCAGCTCAGA |
| Runx2 | GATCTGAGATTTGTGGGCCGGA | ATGGGGATGTCATCTGGCTCAG |
| JIP1 | AAAGGCAGGCCCAGTTGGTG | TGTCATGGGTGAGCCAGTGCT |
| JIP2 | CGGGCTGTCTTCAGGTTCATCC | GTGCGCATGTTGAAGCCACG |
| JIP3 | CAGCTCCAGCTACCAGTGTCC | GCTCTCGTTGAGTGGCGTGA |
| JIP4 | TGTAGCCCAGTGGAGGAAATGT | TGCATCGTATGGAATGATGAGG |

Primer sequences for qRT-PCR.

Table S6

Antibodies used in this article.

| Antibodies | Source | Catalog |
| --- | --- | --- |
| Mouse Anti-HSP90 | Santa Cruz | sc-13119 |
| Rabbit Anti-RUNX2 | CST | 8486 |
| Rabbit Anti-OSX | Abcam | ab209484 |
| Rabbit Anti-SIRT5 | Proteintech | 15122-1-AP |
| Mouse Anti-SIRT5 | Proteintech | 67257-1-Ig |
| Rabbit Anti-NFATC1 | CST | 8032 |
| Rabbit Anti-MITF | CST | 97800 |
| Rabbit Anti-PU.1 | CST | 2258 |
| Rabbit Anti-CTSK | Proteintech | 11239-1-AP |
| Rabbit Anti-TOM20 | CST | 42406 |
| Rabbit Anti-TRAP | Abcam | ab133238 |
| Rabbit Anti-ATP5A1 | Proteintech | 66037-1-Ig |
| Rabbit Anti-OGDH | CST | 26865 |
| Rabbit Anti-JIP4 | CST | 5519 |
| Rabbit Anti-P38 | CST | 8690 |
| Rabbit Anti-p-JNK | CST | 4668 |
| Rabbit Anti-p-P38 | CST | 4511 |
| HRP-conjugated GAPDH | Proteintech | HRP-60004 |
| HRP-linked Anti-Mouse | CST | 7076 |
| HRP-linked Anti-Rabbit | CST | 7074 |

Figure S1


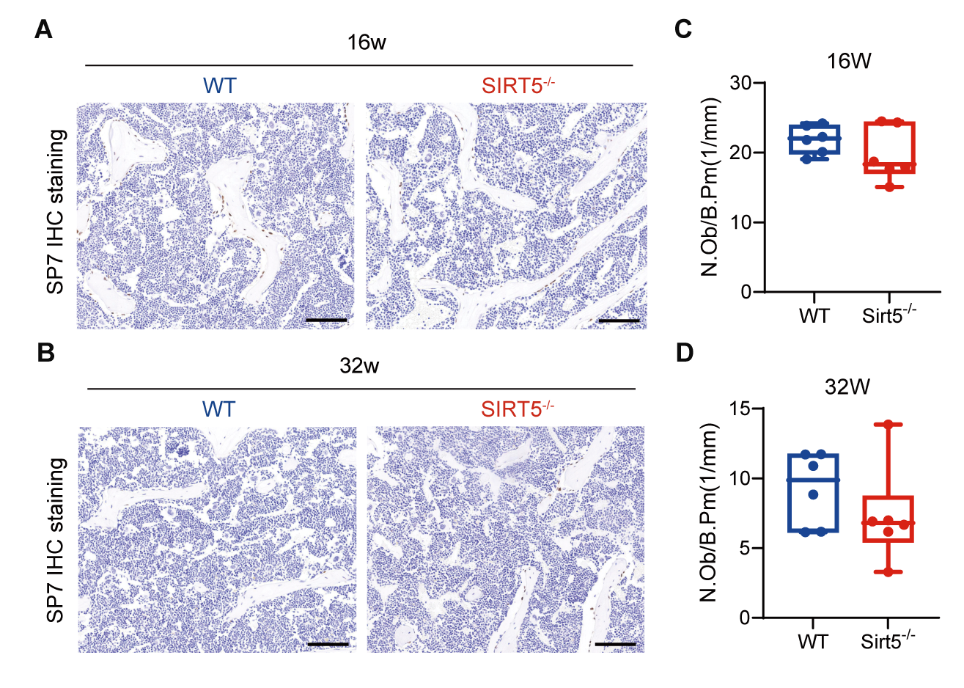


**(A-B)** Representative images of SP7 immunohistochemical staining of distal femoral epiphyses from 16- and 32-week-old WT and *Sirt5*^-/-^ mice. Scale bar, 100 μm. **(C-D)** The quantification of osteoblast number/bone trabecular perimeter (N.Ob/B.Pm) (*n* = 6). The data are presented as means ± SD.

Figure S2


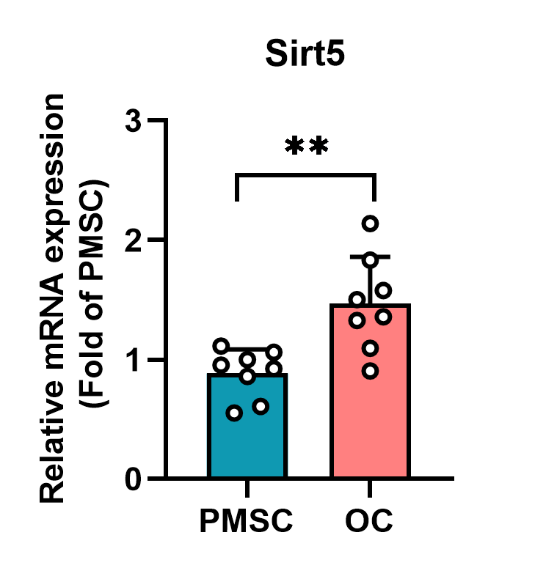


Relative mRNA expression of *Sirt5* in human osteoclast (OC)-like cells and their precursor peripheral blood mononuclear cells (PBMC) (n = 8). The data were extracted from the literature (doi:10.3390/genes14040916). The data are presented as means ± SD. **p < 0.01 vs. PBMC.

Figure S3


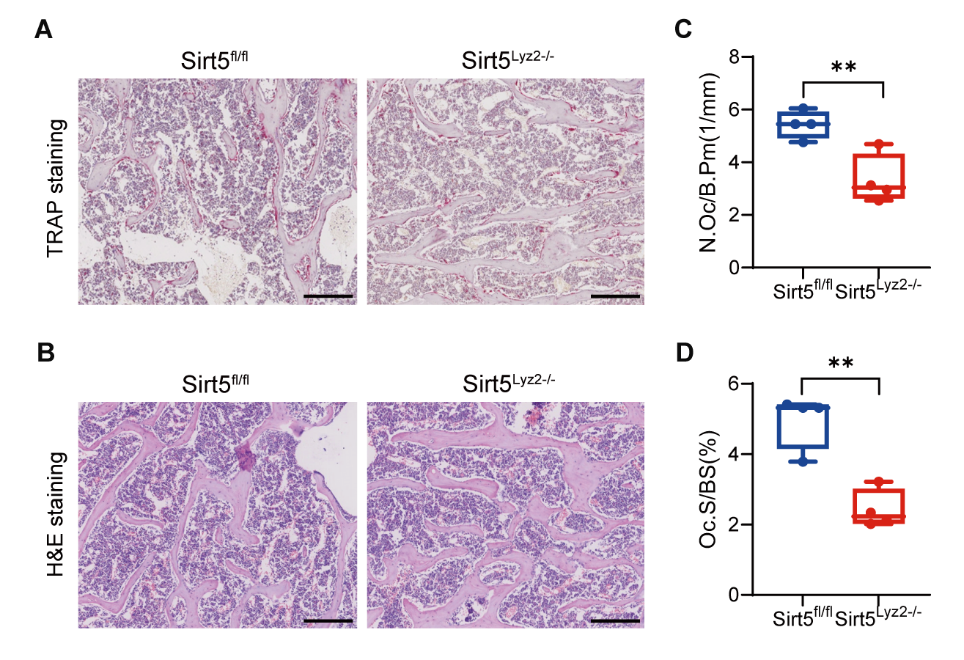


**(A-B)** Representative images of TRAP (A) and H&E staining (B) of lumbar vertebrae from *Sirt5*^fl/fl^ and *Sirt5*^Lyz2-/-^ mice. Scale bar, 200 μm. **(C)** The quantification of osteoclast number/bone trabecular perimeter (N.Oc/B.Pm) (*n* = 4). **(D)** The quantification of osteoclast surface/bone trabecular surface (Oc.S/BS) (*n* = 4). The data are presented as means ± SD. **p < 0.01 vs. Sirt5^fl/fl^.

Figure S4


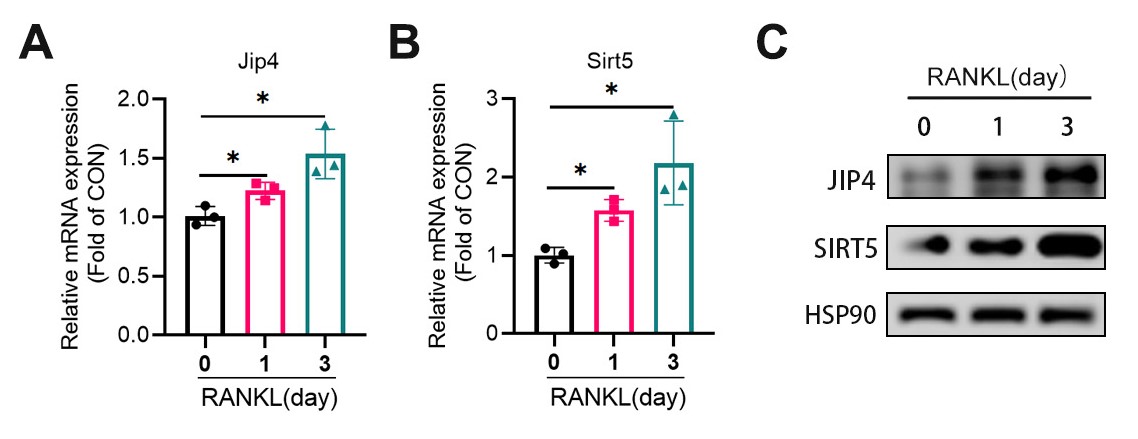


**(A-C)** The expression of SIRT5 and JIP4 at the gene levels(A-B) and protein levels(C) after RANKL stimulation for 0, 1, or 3 days. The data are presented as means ± SD. *p < 0.05 vs. day 0.
